# Autogenic-regenerated intestinal transplantation improves outcomes in short bowel syndrome

**DOI:** 10.1172/jci.insight.200275

**Published:** 2026-06-02

**Authors:** Kentaro Iwaki, Takamichi Ishii, Hidenobu Kojima, Fumiaki Munekage, Hiroshi Horie, Kenta Makino, Takuma Karasuyama, Yusuke Hanabata, Elena Yukie Uebayashi, Satoshi Ogiso, Etsuro Hatano

**Affiliations:** Department of Surgery, Graduate School of Medicine, Kyoto University, Kyoto, Japan.

**Keywords:** Development, Gastroenterology, Organ transplantation, Transplantation

## Abstract

Small bowel transplantation (SBT) is the only curative treatment for intestinal failure due to short bowel syndrome (SBS); however, the 10-year graft survival rate after SBT remains below 50%. Therefore, alternative treatments are required. We developed a potentially new therapeutic strategy for intestinal failure involving in vivo intestinal regeneration using a decellularized scaffold in a rat model. A 3 cm segment of decellularized small intestine was anastomosed to the jejunum for in vivo regeneration. After 4 weeks of regeneration, the entire native intestine was resected to induce SBS, and the regenerated intestine was transplanted into the same rat. Histological analysis revealed regeneration of mucosa, nerves, muscular layer, and crypts, consistent with autologous cell infiltration. An indocyanine green test confirmed blood flow from the adjacent mesentery into the regenerated intestine. The regenerated intestine exhibited absorption of nutrients in vivo, and ex vivo assessments confirmed peristalsis and absorptive capacity comparable with native intestine. Transplantation of the regenerated intestine significantly improved postoperative nutritional status in SBS rats. Our method, autogenic-regenerated intestinal transplantation, showed the therapeutic potential for intestinal failure. This is the first study to our knowledge to demonstrate a functionally integrated regenerated intestine, providing a foundation for future regenerative therapy.

## Abstract 2

Transplantation of a decellularized graft successfully regenerated a functional intestine in vivo, rescuing severe malnutrition in rat short bowel syndrome.

## Graphical abstract

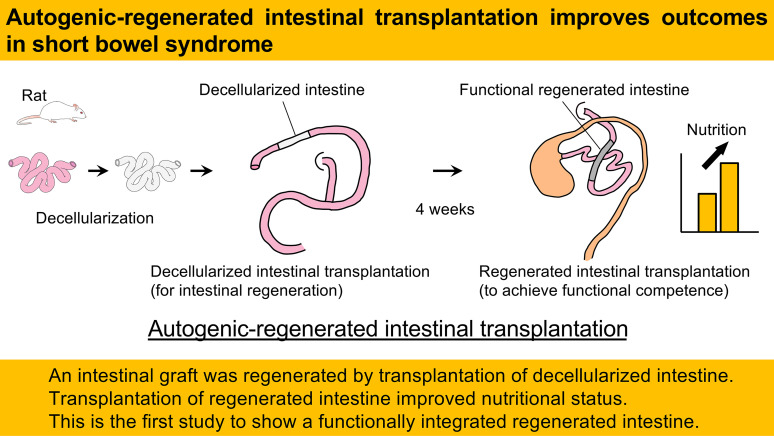

## Introduction

The incidence of intestinal failure, commonly caused by short bowel syndrome (SBS), is estimated to be 20 in 1 million population (1–3). SBS is triggered by congenital intestinal disorders, inflammatory bowel disease, ischemic conditions, tumors, or occurs as a consequence of surgery (4–6). Patients with SBS lose intestinal autonomy and suffer from severe malnutrition because of their lack of absorption capacity, which necessitates parenteral nutrition (3, 7, 8). In this setting, complications, including life-threatening infections, thrombosis, liver failure, and developmental disorders in children, reduce patient quality of life and increasing healthcare costs (1, 7, 9). Although the only curative treatment for intestinal failure is small-bowel transplantation (SBT) (4–6), the 10-year graft survival rate after SBT remains below 50% because of the high allograft rejection rate (10–12). Therefore, alternative treatments are required for intestinal failure.

In response, various approaches have been explored to reconstruct artificial intestines, including the use of intestinal organoids and induced pluripotent stem cells (iPSCs) (13–18). However, the reconstruction of various intestinal components, including the mucosa, muscular layer, enteric nervous system, and vasculature, as a tubular organ remains challenging. To date, no engineered intestine has achieved structural and functional integration in vivo.

In recent years, decellularization technology has been developed to remove cellular components from organs while preserving their 3-dimensional ultrastructure and extracellular matrix (13, 19–21). Two-dimensional decellularized scaffolds have already been clinically applied included in decellularized small intestinal submucosa, nerves, urinary bladder and heart valves. However, 3-dimensional functional organs utilizing decellularized scaffolds have not yet been achieved (22–25). We have previously reported in vivo regeneration of a small intestinal graft from a decellularized scaffold (26). By anastomosing the decellularized intestine with the native intestine, we successfully regenerated a well-organized tubular intestine using autologous cells in vivo (26). However, it did not address whether the regenerated intestine could exhibit functional capacity or exert therapeutic benefit in a clinically relevant disease model.

In this study, we advance this concept from proof-of-structure to proof-of-function and therapeutic relevance. We transplanted autogenic-regenerated intestines into the alimentary tract of SBS model rats and evaluated both their physiological functions and their effects on survival and nutritional status. This work represents the first study to our knowledge demonstrate that an in vivo–regenerated intestine can functionally integrate and improve outcomes in SBS model rat, laying the foundation for future translation of regenerative therapy for intestinal failure.

## Results

### Decellularization of rat small intestine.

**Figure 1:**
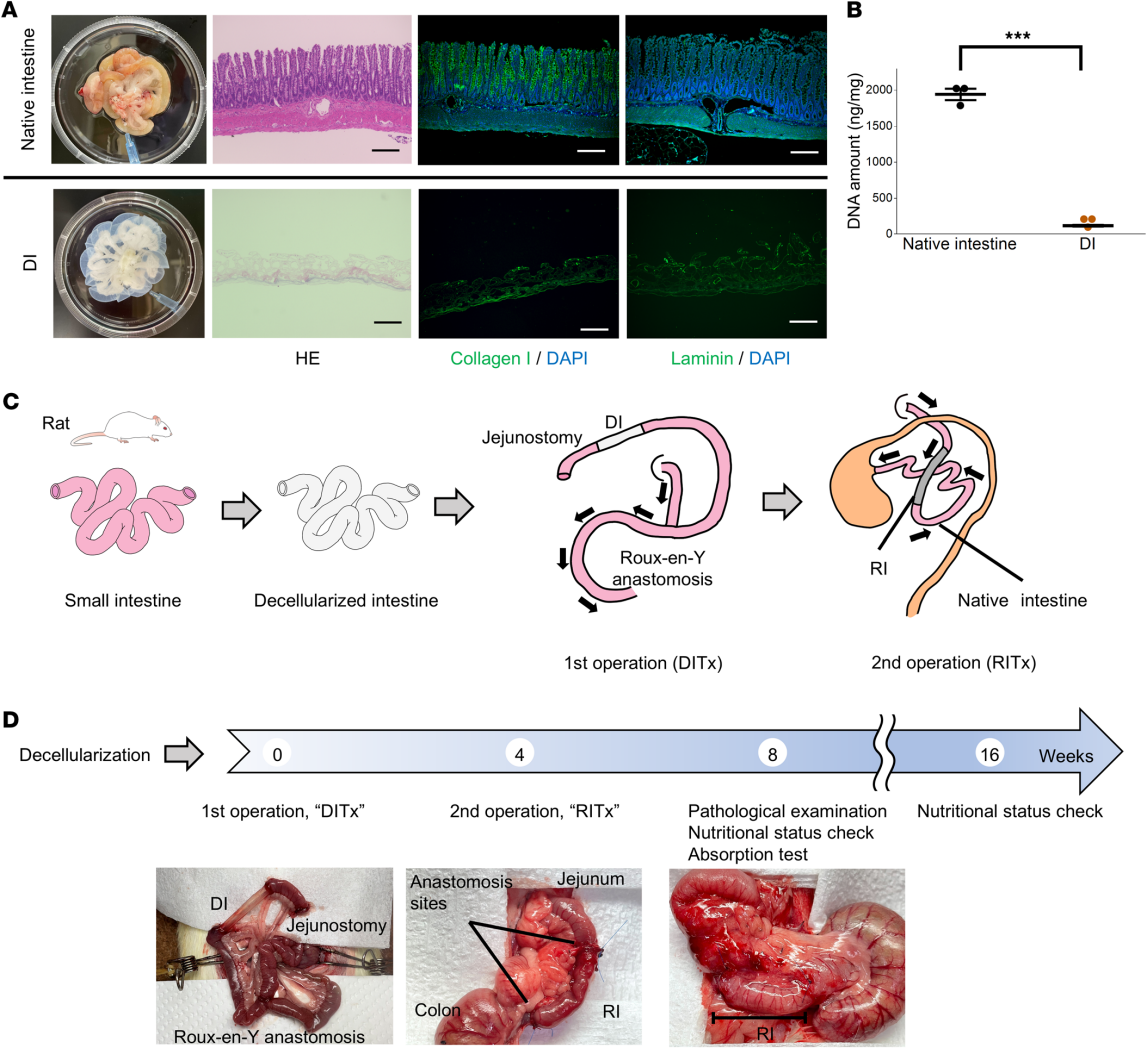
Intestinal decellularization, schematic illustrations of autogenic-regenerated intestinal transplantation and the study timeline. (**A**) Macroscopic images, H&E staining and immunofluorescence images of the native and decellularized intestines. Scale bar: 200 μm. (**B**) Quantification of DNA in the native and decellularized intestine. *n* = 3 for each group. (**C**) Schematic illustration of autogenic-regenerated intestinal transplantation. In the first operation, DITx, the decellularized intestine was transplanted into the elevated jejunum to promote in vivo organ regeneration. At 4 weeks after DITx, in the second operation of RITx, the regenerated intestine was transplanted into the main alimentary tract to exert its function. Arrows indicate the direction of dietary passage. (**D**) The study timeline. Student’s *t* test (**B**). ***P < 0.001. DI, decellularized intestine; DITx, decellularized intestinal transplantation; H&E, hematoxylin and eosin; RI, regenerated intestine; RITx, regenerated intestinal transplantation.

Decellularization of a rat small intestine was performed as previously described (20, 26–29). After procurement of a whole small intestine, the intestine was frozen at –80°C and thawed overnight at 4°C. Subsequently, decellularization was performed with 0.25 w/v% trypsin and 1 mmol/L ethylenediaminetetraacetic acid (EDTA) solution at 37°C for 1 hour, followed by 0.1% Triton X/0.05% EDTA solution for 24 hours. Histological analysis revealed that the decellularized intestine retained its extracellular matrix (Figure 1A) and was devoid of cells, reducing its DNA (native intestine: 1,943.9 ± 79.2 ng/mg versus decellularized intestine: 1,14.2 ± 11.1 ng/mg, *P* < 0.001, *n* = 3) (Figure 1B).

### Evaluations of the intestinal regeneration process.

Autogenic-regenerated intestinal transplantation consisted of 2 operations (Figure 1C). After transection of the proximal jejunum, a Roux-en-Y anastomosis was performed. A 3 cm segment of the native small intestine was removed from the elevated jejunum. Then, a 3 cm segment of the decellularized intestine, corresponding to approximately 5% of the rat small intestine, with a silicon tube as an internal stent was anastomosed to the elevated jejunum with 6-0 VICRYL running sutures (decellularized intestinal transplantation, DITx). In the second operation, regenerated intestinal transplantation (RITx) was performed 4 weeks post-DITx. The regenerated intestine was identified by using an internal stent and marked sutures. After isolating the regenerated intestine with its mesentery, it was anastomosed to the jejunum and ileum using 6-0 VICRYL running sutures. Thereafter, the dietary content passed through the regenerated intestine. Both ends of the regenerated intestine were marked with nonabsorbable sutures (Figure 1, C and D, and Supplemental Figure 1; supplemental material available online with this article; https://doi.org/10.1172/jci.insight.200275DS1).

**Figure 2:**
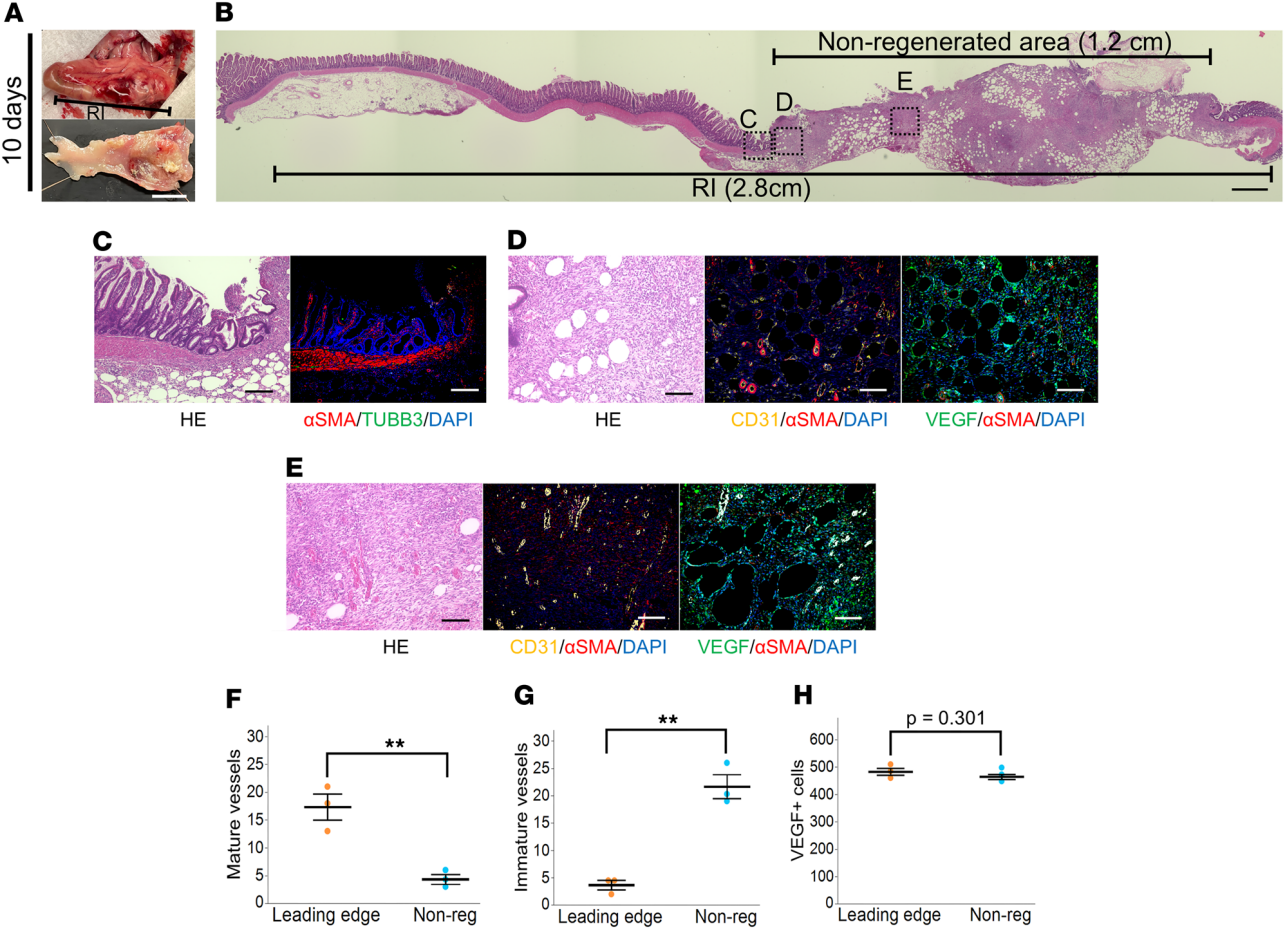
Evaluation of the intestinal regeneration process in 10 days after DITx. (**A**) Macroscopic images of the regenerated intestine 10 days after DITx. Scale bar: 1 cm. (**B**) H&E staining of the regenerated intestine 10 days after DITx. (**C**) H&E staining and immunofluorescence images (αSMA/TUBB3) of the leading edge of the regenerated area. (**D**) H&E staining and immunofluorescence images (CD31/αSMA and VEGF/αSMA) of the leading edge of the regenerated area. (**E**) H&E staining and immunofluorescence images (CD31/αSMA and VEGF/αSMA) of the nonregenerated area. Scale bars: 1,000 μm (**B**), 200 μm (**C**), and 100 μm (**D** and **E**). (**F**) The number of mature vessels (CD31^+^ and αSMA^+^) per high-power field in the leading edge of regenerated area and nonregenerated area. *n* = 3 for each group. (**G**) The number of immature vessels (CD31^+^ and αSMA^–^) per high-power field in the leading edge of regenerated area and nonregenerated area. *n* = 3 for each group. (**H**) The number of VEGF^+^ cells per high-power field in the leading edge of the regenerated area and nonregenerated area. *n* = 3 for each group. Student’s *t* test (**F**–**H**). **P < 0.01. αSMA, α-smooth muscle actin; DAPI, 4′,6-diamidino-2-phenylindole; DITx, decellularized intestinal transplantation; Non-reg; nonregenerated area; RI, regenerated intestine; TUBB3, tubulin β 3; VEGF, vascular endothelial growth factor.

Macroscopic observation of the decellularized intestine 10 days after DITx revealed a mucosal defect (Figure 2A). H&E staining showed that the implanted decellularized intestine and nonregenerated region lacking mucosa and muscular layers were 2.8 cm and 1.2 cm long, respectively (Figure 2B). At the leading edge of the regenerated area, the mucosa, followed by the muscle layer, infiltrated the decellularized intestine, which was filled with fibroblasts, myofibroblasts, and inflammatory cells (Figure 2, C and D). CD31 staining revealed the presence of blood vessels in the leading edge of the regenerated area as well as in the more distal nonregenerated area (Figure 2, D and E). Mature blood vessels identified by CD31^+^ and αSMA^+^ vessels were significantly more frequent at the leading edge of the regenerated area than in the nonregenerated area (17.3 ± 4.0 versus 4.3 ± 1.5, *P* = 0.007, *n* = 3) (Figure 2F), whereas the nonregenerated area contained significantly more immature blood vessels identified by CD31^+^ and αSMA^–^ vessels (21.7 ± 3.8 versus 3.7 ± 1.5, *P* = 0.002, *n* = 3) (Figure 2G) (30–32). Furthermore, both the leading edge of the regenerated area and the nonregenerated area exhibited high levels of VEGF^+^ cells (Figure 2, D, E, and H). These findings suggest that the decellularized intestine is initially filled with fibroblasts, myofibroblasts, and inflammatory cells followed by angiogenesis and that mucosal and muscle layer are regenerated from the margins.

**Figure 3:**
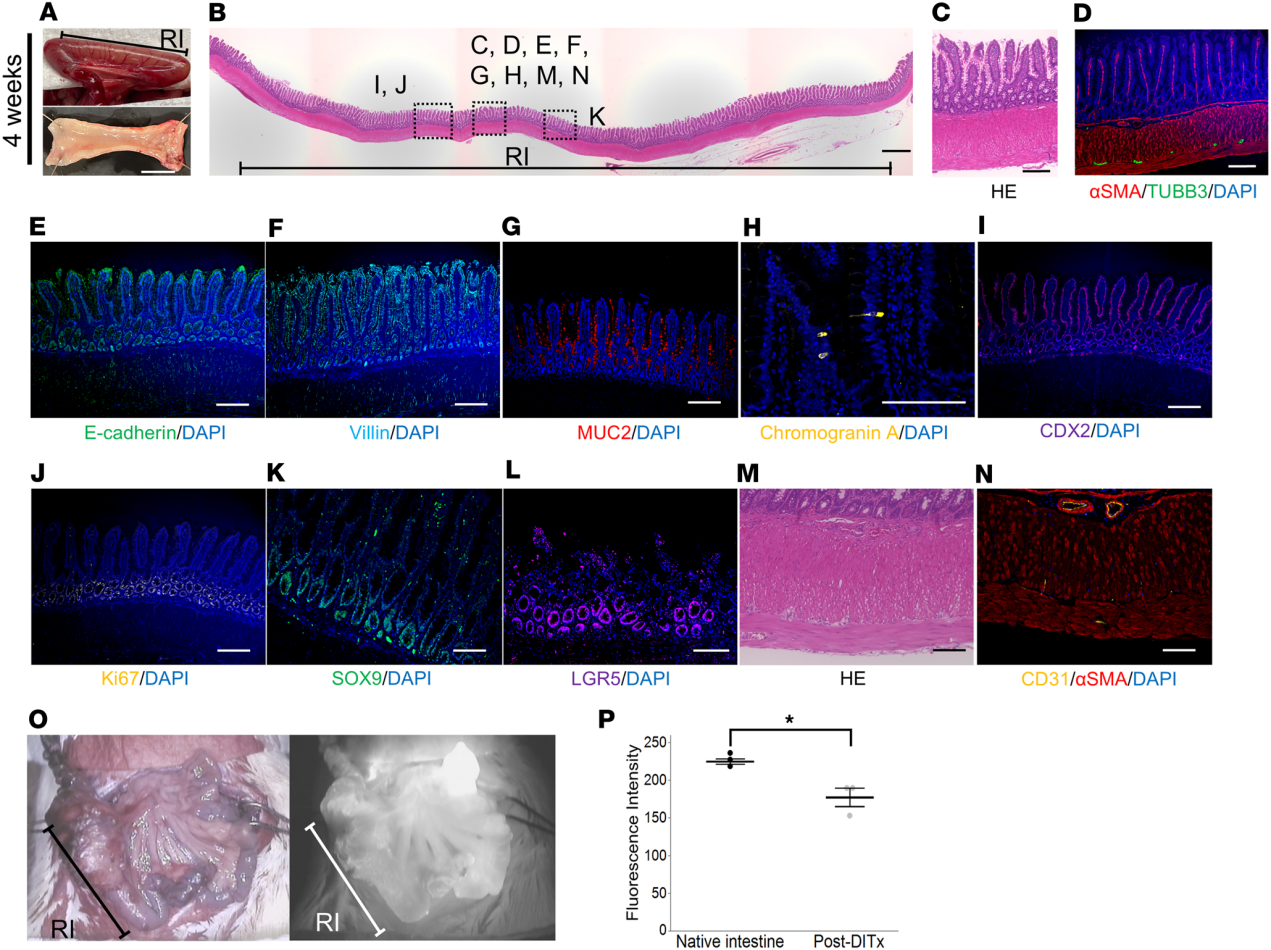
Histological evaluations of the regenerated intestine 4 weeks after DITx. (**A**) Macroscopic images of the regenerated intestine 4 weeks post-DITx. Scale bar: 1 cm. (**B**) H&E staining of the regenerated intestine 4 weeks post-DITx. Scale bars: 1000 μm. (**C**) High-magnification H&E staining. Scale bar: 200 μm. (**D**–**L**) Immunofluorescence images showing the expression of (**D**) αSMA/TUBB3, (**E**) E-cadherin, (**F**) villin, (**G**) MUC2, (**H**) chromogranin A, (**I**) CDX2, (**J**) Ki67, (**K**) SOX9 and (**L**) LGR5. (**M** and **N**) H&E staining and immunofluorescence images for CD31/αSMA of a submucosal vessel in the regenerated intestine. Scale bars: 200 μm (**D**–**G**, **I**, **J**, and **L**), and 100 μm (**H**, **K**, **M**, and **N**). (**O**) Intraoperative ICG fluorescence imaging of the regenerated intestine post-DITx. (**P**) Fluorescence intensity of the native and regenerated intestine. *n* = 3 for each group. Student’s *t* test (**P**). *P < 0.05. αSMA, α-smooth muscle actin; DITx, decellularized intestinal transplantation; ICG, indocyanine green; RI, regenerated intestine; TUBB3, tubulin β 3.

Macroscopic observation of the decellularized intestine 4 weeks after DITx revealed angiogenesis from marginal vessels in the adherent mesentery and no mucosal defect (Figure 3A). Luminal stricture was not observed in the decellularized scaffold 4 weeks after DITx. This may be attributed to the use of a stent in the scaffold. Although the mucosal layer in the decellularized intestine was comparatively thin, the mucosa, submucosa, muscular layer, and serosa covered the entire decellularized intestine 4 weeks after DITx (Figure 3, B and C). Immunofluorescence staining of αSMA and TUBB3 revealed that neurons were reconstructed in the submucosal and muscular layers (Figure 3D). The expression of E-cadherin and villin indicated that intercellular adhesion was reconstituted in the regenerated epithelium (Figure 3, E and F). The existence of MUC2^+^ and chromogranin A^+^ cells suggested the presence of goblet cells and enteroendocrine cells (Figure 3, G and H). The regenerated intestine exhibited abundant CDX2, Ki67, SOX9, and LGR5, indicating active epithelial regeneration and proliferation (Figure 3, I–L). Mature blood vessels, evidenced by CD31^+^ and αSMA^+^, were observed in the submucosal layer of the regenerated intestine (Figure 3, M and N). Fluorescence images were obtained following indocyanine green (ICG) injection to examine the blood flow in the regenerated intestine. The ICG test demonstrated that blood flow was supplied from the adjacent mesentery to the regenerated intestine (Figure 3O and Supplemental Video 1). Blood flow into the regenerated intestine was maintained even after the resection of both ends of the regenerated intestine (Supplemental Video 1). Although the fluorescence intensity of the regenerated intestine was significantly lower than that of the native intestine (*P* = 0.020) (Figure 3P), the above findings indicate angiogenesis from the mesentery to the regenerated intestine.

### Histological evaluations of the regenerated intestine post-RITx.

At 4 weeks after DITx, the regenerated intestine was isolated, along with its mesentery, and transplanted into the main alimentary tract (RITx), enabling the passage of food through the regenerated intestine (Figure 1D and Supplemental Figure 1). The rats survived without any complications after these procedures.

**Figure 4:**
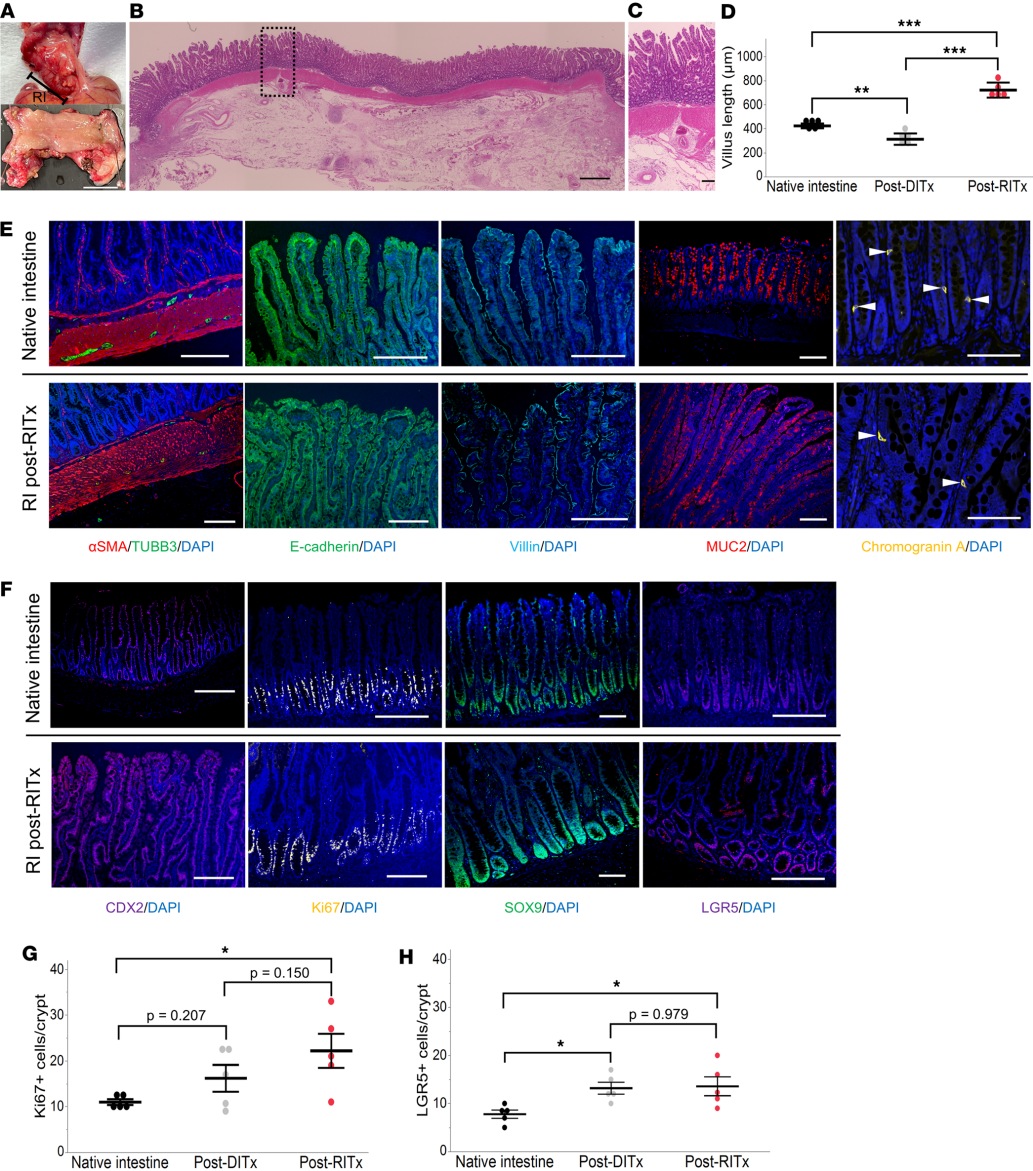
Histological evaluations of the regenerated intestine 4 weeks after RITx. (**A**) Macroscopic images of the regenerated intestine 4 weeks after RITx. Scale bar: 1 cm. (**B** and **C**) H&E staining of the regenerated intestine. Scale bars: 1000 μm (**B**) and 200 μm (**C**). (**D**)Villus length of the native and regenerated intestines. *n* = 5 for each group. (**E**) Immunofluorescence images showing the expression of αSMA, TUBB3, E-cadherin, villin, MUC2, and chromogranin A in the native and regenerated intestines. Scale bar: 200 μm (αSMA/TUBB3, E-cadherin, villin, MUC2) and 100 μm (chromogranin A). (**F**) Immunofluorescence images showing the expression of CDX2, Ki67, SOX9, and LGR5 in the native and regenerated intestines. Scale bars: 200 μm (CDX2, Ki67, LGR5) and 100 μm (SOX9). (**G**) Ki67^+^ cell count per crypt. *n* = 5 for each group. (**H**) LGR5^+^ cell count per crypt. *n* = 5 for each group. One-way ANOVA followed by Tukey’s HSD test (**D**, **G**, and **H**). *P < 0.05. **P < 0.01. ***P < 0.001. αSMA, α-smooth muscle actin; DITx, decellularized intestinal transplantation; RI, regenerated intestine; RITx, regenerated intestinal transplantation; TUBB3, tubulin β 3.

Macroscopic imaging of the regenerated intestine 4 weeks after RITx showed a thick, well-developed mucosa (Figure 4A). H&E staining of the regenerated intestine demonstrated abundant mucosa, submucosa, muscular layer, serosa, and vessels (Figure 4, B and C). Villus length in the regenerated intestine after DITx was significantly shorter than that in the native intestine (314 ± 21.3 μm versus 424 ± 8.9 μm, *P* = 0.007, *n* = 5); however, the villus length after RITx was significantly longer than that of the native intestine (723 ± 27.8 μm versus 424 ± 8.9 μm, *P* < 0.001, *n* = 5) (Figure 4D). αSMA and vimentin^+^ cells were detected in the lamina propria of the mucosa, indicating the presence of intestinal subepithelial myofibroblasts (Figure 4E and Supplemental Figure 2), which contribute to epithelial stem cell growth (33, 34). Immunostaining for TUBB3 revealed the nerve plexus in the submucosa and muscular layers (Figure 4E). E-cadherin^+^ and villin^+^ epithelia were observed throughout the villi in the regenerated intestine as well as in the native intestine, suggesting the maturation of villi and the presence of intestinal epithelial barrier function (Figure 4E). Goblet and enteroendocrine cells were identified by MUC2 and chromogranin A in the regenerated intestine, indicating the regeneration of the well-developed epithelium (Figure 4E). Expression of CDX2, Ki67, SOX9, and LGR5 was observed in the regenerated intestine after RITx (Figure 4F). The numbers of Ki67^+^ (22.2 ± 8.3 versus 11.0 ± 1.4, *P* = 0.014, *n* = 5) (Figure 4G) and LGR5^+^ cells (13.6 ± 2.0 versus 7.8 ± 1.9, *P* = 0.035, *n* = 5) (Figure 4H) were significantly higher in the regenerated intestine after RITx than in the native intestine. Thus, while the regenerated intestine acquired maturity and maintained homeostasis, the components in the regenerated intestine differentiated and proliferated more actively than those in the native intestine.

### Functional analyses of the regenerated intestine after RITx.

**Figure 5:**
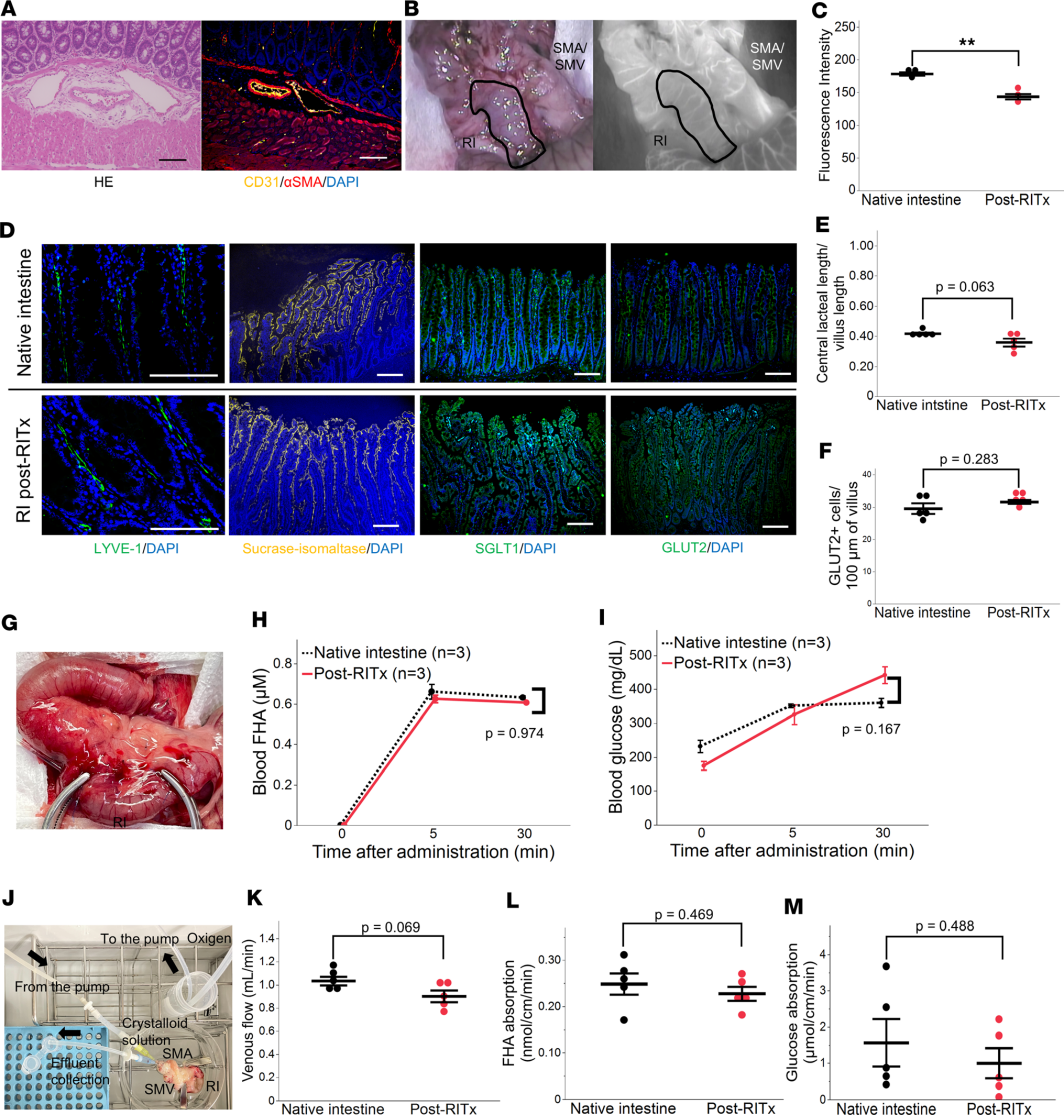
Functional analyses of the regenerated intestine 4 weeks after RITx. (**A**) H&E and immunofluorescence images (CD31/αSMA) in the regenerated intestine 4 weeks after RITx. (**B**) Intraoperative ICG fluorescence imaging of the regenerated intestine. (**C**) Fluorescence intensity of the native and regenerated intestine. *n* = 3 for each group. (**D**) Immunofluorescence images showing the expression of LYVE-1, sucrase-isomaltase, SGLT1, and GLUT2 of the native and regenerated intestine. Scale bars: 200 μm (sucrase-isomaltase) and 100 μm (LYVE-1, SGLT1, and GLUT2). (**E**) The ratio of central lacteal length to villus length per field of view. *n* = 5 for each group. (**F**) The number of GLUT2^+^ cells per 100 μm of villus. *n* = 5 for each group. (**G**) The in vivo absorption test. (**H**) The blood FHA level in the regenerated and native intestines after injection. (**I**) The blood glucose level in the regenerated and native intestines after injection. (**J**) The ex vivo absorptive test. (**K**) The venous flow of the regenerated and native intestines. *n* = 5 for each group. (**L**) The FHA levels in the perfusate from the regenerated and native intestines. *n* = 5 for each group. (**M**) The glucose levels in the perfusate from the regenerated and native intestines. *n* = 5 for each group. Student’s *t* test (**C**, **E**, **F**, and **K**–**M**). A linear mixed-effects model with fixed effects of group, time, and their interaction, and a random effect for individual rats with an AR(1) covariance structure for repeated measures. *P* values were derived from the fixed effects (**H** and **I**). **P < 0.01. αSMA, α-smooth muscle actin; FHA, 6-Fluorescein-5(6)-carboxamido hexanoic acid; GLUT2, glucose transporter 2; ICG, indocyanine green; LYVE-1, lymphatic vessel endothelial hyaluronan receptor-1; RI, regenerated intestine; RITx, regenerated intestinal transplantation; SGLT1, sodium-glucose linked transporter 1; SMA, superior mesenteric artery; SMV, superior mesenteric vein.

Mature blood vessels positive for CD31 and αSMA were observed in the submucosal layer of the regenerated intestine after RITx (Figure 5A). The ICG test confirmed the maintenance of blood flow from the mesentery into the regenerated intestine 4 weeks after RITx (Figure 5B). Although the fluorescence intensity in the regenerated intestine after RITx was still lower than that in the native intestine (*P* = 0.002) (Figure 5C), the presence of blood flow in the regenerated intestine suggests its potential for nutrient absorption.

We then evaluated the absorption ability of regenerated intestines. Central lacteals identified by LYVE-1 staining were similarly developed in the regenerated and native intestine (Figure 5, D and E). Regarding sugar absorption, sucrase-isomaltase, which is involved in the hydrolysis of disaccharides, sodium-glucose linked transporter 1 (SGLT1), and glucose transporter 2 (GLUT2), were expressed in the regenerated intestine (Figure 5D). The number of GLUT2^+^ cells in the regenerated intestine was equivalent to that in the native intestine (31.6 ± 1.3 versus 29.6 ± 3.6, *P* = 0.283, *n* = 5) (Figure 5F).

We conducted in vivo evaluations of the absorption capacity. Both ends of the regenerated intestine 4 weeks after RITx were identified and clamped (Figure 5G). We injected 850 μM fluorescence-labeled medium-chain fatty acids [6-Fluorescein-5(6)-carboxamido hexanoic acid; FHA] and 25% glucose into the regenerated intestinal lumen and evaluated blood levels. FHA was absorbed via passive diffusion from the gastrointestinal tract to the capillaries without passing through the lymph vessels (13, 35). Blood FHA and glucose levels significantly increased in a time-dependent manner after the injection in both the regenerated (*P* < 0.001 and *P* < 0.001, respectively) and native intestines (*P* = 0.006 and *P* < 0.001, respectively) (Figure 5, H and I). There was no significant difference in the blood levels between the 2 groups (Figure 5, H and I). These results indicate that the regenerated intestine after RITx had absorptive capability similar to that of the native intestine.

In vivo experiments might be affected by circulating blood volume and glucose tolerance in individual rats. Therefore, we conducted ex vivo absorption tests to verify the absorptive capability of the grafts. After removal of regenerated or native intestines with the superior mesenteric artery (SMA) and superior mesenteric vein (SMV), FHA and glucose were injected into the intestinal lumen. Ex vivo perfusion was performed via the SMA and the perfusate was collected from the SMV (Figure 5J). There was no significant difference in the venous flow between the regenerated and native intestines (0.9 ± 0.1 mL/min versus 1.0 ± 0.1 mL/min, *P* = 0.068, *n* = 5) (Figure 5K), and almost all perfused liquid was collected, suggesting no major leakage from the removed intestines and vessels. No significant difference was found in the FHA (0.23 ± 0.03 nmol/cm/min versus 0.25 ± 0.05 nmol/cm/min, *P* = 0.469, *n* = 5) and glucose levels (1.0 ± 0.9 μmol/cm/min versus 1.6 ± 1.5 μmol/cm/min, *P* = 0.488, *n* = 5) between the regenerated and native intestines (Figure 5, L and M). These results show that the absorptive capability of the regenerated intestine post-RITx was comparable with that of the native intestine.

### Evaluations of peristalsis.

**Figure 6:**
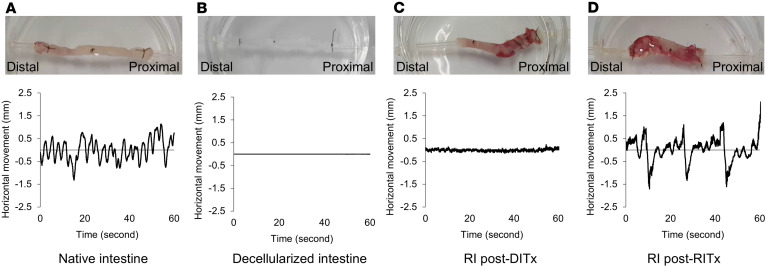
Evaluation of peristalsis. (**A**–**D**) Macroscopic images (upper panels) and time-displacement plots (lower panels, *x* axis: seconds, *y* axis: horizontal movement of a marked point at the center of the intestine) of intestinal peristalsis. (**A**) Native intestine. (**B**) Decellularized intestine. (**C**) Regenerated intestine 4 weeks after DITx. (**D**) Regenerated intestine 4 weeks after RITx. *n* = 3 for each group. The right end represents the proximal side and the left end the distal side. The waveforms were created from a representative example (Supplemental Video 2). The plots depict spatiotemporal displacement of a single tracked point. DI, decellularized intestine; DITx, decellularized intestinal transplantation; RI, regenerated intestine; RITx, regenerated intestinal transplantation.

Both ends of the intestines were fixed to connectors, and their peristalsis was recorded. Horizontal movement of the grafts was analyzed to create a time-displacement plot. We confirmed that the native intestine exhibited normal peristalsis and that the decellularized intestine showed no peristalsis (Figure 6, A and B, and Supplemental Video 2). While the regenerated intestine 4 weeks after DITx showed extremely weak peristalsis, the regenerated intestine 4 weeks after RITx exhibited periodic peristaltic waves, similarly to those of the native intestine. We confirmed that the antegrade peristaltic waves propagated from the proximal to the distal side (Figure 6, C and D, and Supplemental Video 2). Thus, the regenerated intestine possesses motility functions, in addition to its absorptive capacity.

### Nutritional status evaluations after RITx in SBS model rats.

**Figure 7:**
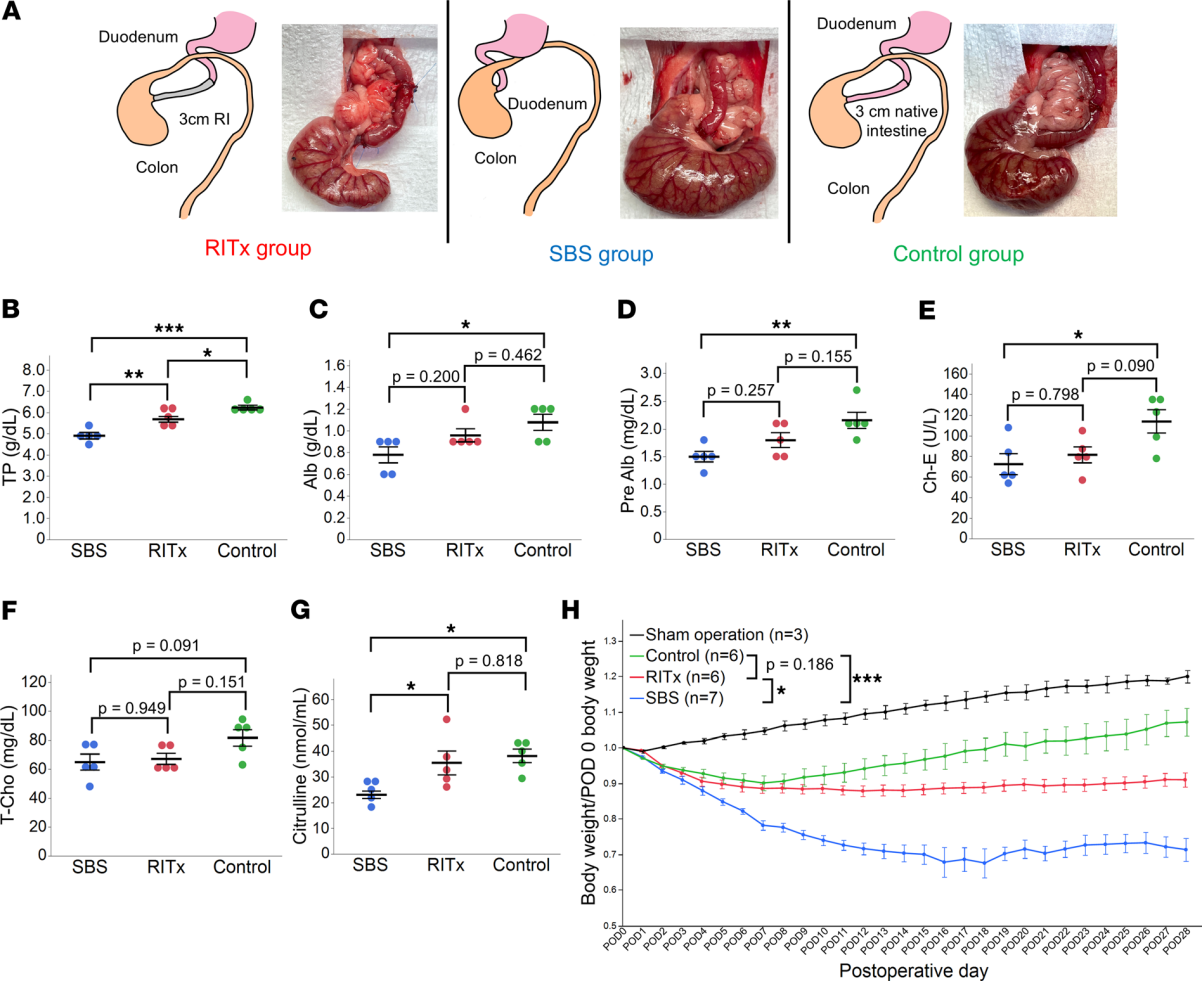
Nutritional status evaluations after RITx in disease models. (**A**) Schematic illustrations and intraoperative images of the 3 groups. In the RITx group (left), the entire native intestine was resected, and 3 cm of regenerated intestine was anastomosed between the duodenum and terminal ileum. In the SBS group (middle), the entire native intestine was resected, and the duodenum was anastomosed directly to the terminal ileum. In the control group (right), 3 cm of native intestine was preserved, and the remaining intestine was resected. Nutritional markers in blood samples from each group on postoperative day (POD) 28. *n* = 5 for each group. (**B**) Total protein. (**C**) Albumin. (**D**) Prealbumin. (**E**) Cholinesterase. (**F**) Total cholesterol. (**G**) Citrulline. (**H**) Changes in the body weight loss rate (body weight/body weight on POD 0). One-way ANOVA followed by Tukey’s (HSD) test (**B**–**G**). A linear mixed-effects model with fixed effects of group, time, and their interaction, and a random effect for individual rats with an AR(1) covariance structure was used, followed by Tukey’s HSD test for post hoc pairwise comparisons (**H**). *P < 0.05, **P < 0.01, ***P < 0.001. Alb, albumin; Ch-E, cholinesterase; POD, postoperative day; Pre Alb, prealbumin; RI, regenerated intestine; RITx, regenerated intestinal transplantation; SBS, short bowel syndrome; T-Cho, total cholesterol; TP, total protein.

To explore the therapeutic effects of autogenic-regenerated intestinal transplantation on intestinal failure, nutritional status was compared among 3 groups in the rat SBS model: the RITx group, in which 3 cm of regenerated intestine 4 weeks after DITx was anastomosed to the duodenum and terminal ileum; the SBS group, in which the entire native intestine was resected; and the control group, in which 3 cm of native intestine was preserved (Figure 7A). One rat in the SBS group died on postoperative day (POD) 18, while the remaining rats survived during the observation period (Supplemental Figure 3). Blood samples were collected on POD 28 to evaluate nutritional markers (Figure 7, B–G). The SBS group exhibited significantly lower levels of total protein (*P* = 0.004), albumin (*P* = 0.025), prealbumin (*P* = 0.008), cholinesterase (*P* = 0.029), and citrulline (*P* = 0.015) compared with the control group, indicating compromised nutritional status. The total protein levels in the RITx group were significantly lower than those in the control group (*P* = 0.037), whereas they were significantly higher than those in the SBS group (*P* = 0.004) (Figure 7B). Although albumin and prealbumin levels tended to increase in the order of SBS, RITx, and control groups, the differences were not statistically significant (Figure 7, C and D). Choline-esterase and total cholesterol levels also tended to increase in the same order (Figure 7, E and F). Citrulline, which is produced mainly in the small intestine, is considered a crucial marker for the functional size of the small intestine in patients with SBT (36–38). The RITx group exhibited significantly higher citrulline levels than the SBS group (*P* = 0.043), and the levels were comparable with those in the control group (*P* = 0.818) (Figure 7G). These findings validated the SBS model, as the SBS group exhibited significantly poorer nutritional status compared with the control group. Furthermore, the RITx group demonstrated improved nutritional parameters compared with the SBS group, indicating the therapeutic potential of regenerated intestinal transplantation.

Postoperative body weight loss rates were evaluated among the 3 groups. Although all rats experienced postoperative weight loss, the control group began to recover by postoperative week 3, and their weight continued to increase thereafter (Figure 7H). In contrast, the SBS group exhibited progressive weight loss for more than 2 weeks postoperatively, with no signs of recovery (Figure 7H). The SBS group exhibited a significantly greater body weight loss compared with the control group (*P* < 0.001). Notably, although the weight in the RITx group did not recover to the preoperative level, their weight loss plateaued around POD 7 and the weight continued to increase slowly (Figure 7H). Although the RITx group also tended to have greater weight loss than the control group (*P* = 0.186), the loss was significantly less than that observed in the SBS group (*P* = 0.020). These results further validated the SBS model and demonstrated the therapeutic benefit of regenerated intestinal transplantation, as reflected by the degree of weight loss — a key indicator of overall nutritional status.

### Long-term outcomes after RITx.

**Figure 8:**
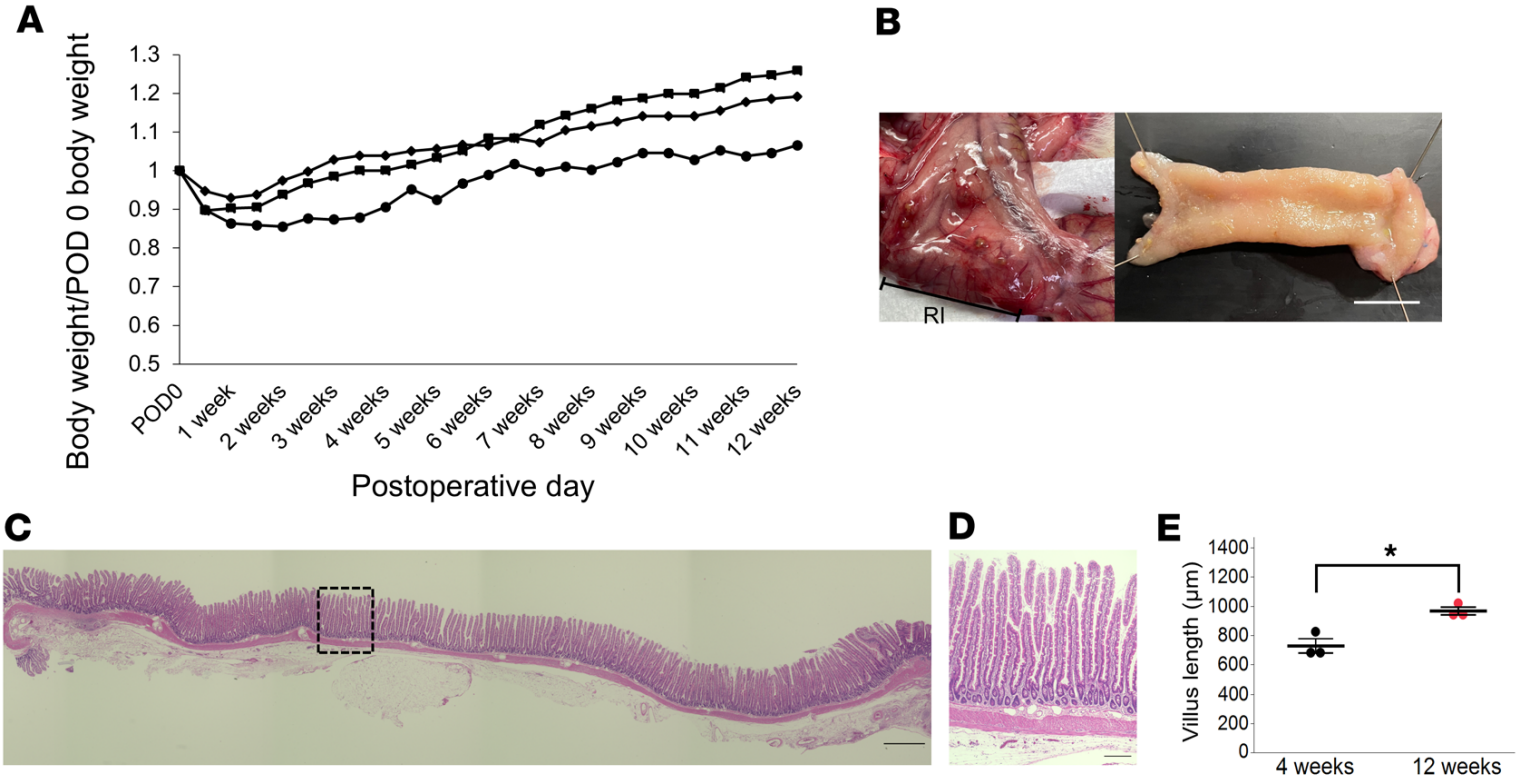
Long-term outcomes in the RITx group. (**A**) Changes in the body weight loss rate (body weight/POD 0 body weight) in 3 rats with RITx for 12 weeks. (**B**) Macroscopic findings of the regenerated intestine at 12 weeks after RITx. Scale bar: 1 cm. (**C** and **D**) H&E staining of the regenerated intestine. Scale bars: 1000 μm (**C**) and 200 μm (**D**). (**E**) Villus length of regenerated intestine 4 and 12 weeks after RITx. *n* = 3 for each group. Student’s *t* test (**D**). *P < 0.05. POD, postoperative day; RI, regenerated intestine; RITx, regenerated intestinal transplantation.

Three rats that underwent RITx were observed for 12 weeks to evaluate the long-term outcomes following autogenic-regenerated intestinal transplantation. No complications were observed during the postoperative course of these rats. Body weight continued to increase and was restored to the preoperative weight between 5 and 9 weeks (Figure 8A). The regenerated intestine was well developed, without malignant tumors or other intestinal disorders (Figure 8B). H&E staining of the regenerated intestine at 12 weeks after RITx showed that the villus length was significantly longer than that observed at 4 weeks after RITx (968 ± 26.0 μm versus 730 ± 48.9 μm, *P* = 0.013, *n* = 3) (Figure 8, C–E).

## Discussion

This study presents the first translational application to our knowledge that bridges structural regeneration and therapeutic efficacy in intestinal tissue regeneration using decellularized scaffolds, which we term “autogenic-regenerated intestinal transplantation.” Initially, this approach regenerated the small intestine from autologous cells using only a decellularized scaffold in vivo. Subsequently, the regenerated intestine was transplanted into the main alimentary tract of the same rat. Autogenic-regenerated intestinal transplantation demonstrated structural and functional regeneration, as well as a significant therapeutic effect on survival and nutritional status in SBS models.

In the process of autogenic regeneration, fibroblasts and inflammatory cells first infiltrate the decellularized scaffold, followed by gradual regeneration of vessels, mucosa, and muscularis from the ends of the decellularized intestine. This result indicates the migration of cells from the adjacent native intestine to the decellularized scaffold, which is similar to the wound-healing process using decellularized scaffolds in other organs (22–24, 39, 40). Notably, H&E staining revealed no histological evidence of fibrotic scarring in the regenerated intestines. In addition, Masson’s trichrome staining revealed that collagen deposition in the regenerated intestine was comparable with that in the native intestine (Supplemental Figure 4). This finding is consistent with previous studies demonstrating in vivo organ regeneration from decellularized scaffolds in the colon and kidney without the need for cell transplantation, similar to our method (39, 40). This may be because the decellularized scaffold retains the microanatomical structures and the extracellular matrix with growth factors.

Although the development of regenerated intestine was still immature after DITx, the regeneration process was promoted after RITx. Previous reports have suggested that dietary passage through the lumen is crucial for the development of tubular organ function (15, 41). Dietary passage after RITx may promote intestinal regeneration and function.

In the mechanism of intestinal regeneration, GLP-2 is an important mediator of intestinal adaptation and epithelial growth and has been clinically used for the treatment of SBS (42–44). Therefore, we measured serum GLP-2 levels to explore whether they contributed to intestinal regeneration in our models (Supplemental Figure 5). There were no significant differences between the groups, suggesting that intestinal regeneration in the present study may not be dependent on serum GLP-2 levels.

In vivo organ regeneration strategies have also been developed using organoids or cell-seeded scaffolds transplanted into the body, utilizing the host as a natural bioreactor (39, 45, 46). While ex vivo organ regeneration has faced challenges such as the difficulty of securing sufficient cell numbers and replicating the complex extracellular environment, in vivo approaches have the potential to overcome these limitations. As a result, in vivo organ regeneration may become a dominant strategy in future organ regeneration. Although further research is needed to clarify the regeneration mechanisms, autogenic-regeneration in vivo from decellularized scaffolds is a promising approach to develop a functional intestine.

Regeneration of intestinal tissue using organoids or iPSCs in a decellularized scaffold has recently been reported to provide an alternative treatment for intestinal failure; however, orthotopic transplantation of the regenerated intestine has not been achieved (13–15, 47). Immediate thrombus formation in the blood vessels is a major problem in artificial organ transplantation (29, 48, 49). Although the blood flow in the regenerated intestine was inferior to that in the native intestine, our method successfully addressed this issue because autogenic regeneration facilitates angiogenesis from the adjacent mesentery. Thus, transplantation of regenerated intestine has the advantage of achieving continuous blood flow in the regenerated intestine, which is crucial for maintaining organ function in vivo.

FHA is absorbed from the surface of villi and transferred to capillaries by passive transport (13, 50). A previous study showed excessive FHA leakage from the decellularized intestine due to insufficient cell concentration (13). In our study, the absorptive capacity of FHA in the regenerated intestine was comparable with that in the native intestine without major leakage. These results indicate that the regenerated intestine might have intestinal epithelial barrier function and reconstructed vascular networks. Furthermore, glucose absorption by active transport was similar between regenerated and native intestines in histological and physiological evaluations. Thus, the regenerated intestine possessed sufficient absorption capabilities for both passive and active transport to improve the outcomes in SBS rats.

The villi of the regenerated intestine were significantly longer than those of the native intestine. Considering the Ki67^+^ and LGR5^+^ cells in the regenerated intestine, villus hypertrophy may reflect the remodeling phase following the regeneration response. Despite villus hypertrophy, the absorption of fatty acids and glucose in the regenerated intestine was comparable with that in the native intestine (Figure 5, H, I, and K–M). This discrepancy might be due to lower blood flow in the regenerated intestine in comparison to that in the native intestine (Figure 3P and Figure 4D). Histological examination at 12 weeks after RITx revealed longer villi than those at 4 weeks after RITx. Given the continuous structural remodeling, vascular maturation and absorptive ability should be investigated over a longer observational period. Further studies are needed to evaluate the relationship between blood flow and absorption.

Another question regarding the clinical applications of this approach is whether DITx can induce intestinal regeneration under SBS conditions. To address this issue, we performed DITx in an SBS model and evaluated the efficacy of autogenic regeneration (Supplemental Figure 6A). The body weight decreased to 76% ± 1.4% after DITx (Supplemental Figure 6B), suggesting that DITx in SBS was highly invasive in small animals without parenteral nutrition. However, near-complete mucosal regeneration (97% ± 0.2%) was observed in the 3 cm graft at 4 weeks after DITx (Supplemental Figure 6, C–F). These findings indicate that, although intestinal regeneration might be partially attenuated under SBS conditions, possibly because systemic nutritional deficiencies limit the metabolic resources available for regeneration, acceptable intestinal regeneration was achieved with DITx, even under SBS conditions. Further studies on DITx in SBS should be performed in large animal models with parenteral nutrition.

Several challenges should be addressed in the clinical application of our method. In the present study, regeneration was induced by anastomosing the decellularized intestine to native intestine at both ends. Food passage in the decellularized scaffold was not allowed to protect anastomotic sites until sufficient regeneration is achieved. This approach might be challenging in patients with severely short bowels due to the limited bowel length. We envision that DITx procedure can be performed using a double-barrel stoma for the clinical application in patients with short bowels (Supplemental Figure 7). This approach might be feasible even in patients with a residual bowel of extremely limited length. In addition, the regenerated intestine can be reconstructed in the alimentary tract simply by stoma closure. The feasibility of this technique is under investigation using porcine model. Scalability is another limitation of the current approach. We attempted to regenerate a 6 cm decellularized intestinal graft, corresponding to approximately 10% of the rat small intestine. Regeneration of a 6 cm graft was incomplete even after 12 weeks after DITx, although the mucosal and muscular layers regenerated almost completely in the decellularized intestine (Supplemental Figure 8). While longer regeneration period might be needed for a 6 cm graft, alternative strategies should be considered to promote regenerative process. One of the potential approaches is a multisegment approach, in which multiple decellularized intestinal segments are implanted and regenerated in parallel. While this approach is difficult to implement in small animal models because of limited abdominal space, it may be feasible in large-animal models. Another potential approach is preconditioning with growth factors or cell seeding in the scaffold. These treatments in the decellularized scaffold may facilitate regeneration in longer grafts (45, 51–53). Meanwhile, because decellularized scaffolds exhibit minimal immunogenicity, xenogeneic decellularized scaffolds from large animals have already been applied in clinical settings, demonstrating the feasibility of their use in humans (45, 54–58). To translate this method into clinical practice, further validation in large animal models is required. Given the established clinical use of xenogeneic decellularized scaffolds, our findings provide a realistic and scalable path toward human application.

In conclusion, we demonstrated that autogenic-regenerated intestinal transplantation using decellularized scaffolds can provide both functional and therapeutic benefits in a rat model of SBS. This approach offers a realistic and potentially paradigm-shifting strategy for the treatment of intestinal failure, bridging the gap between structural regeneration and clinical applicability in regenerative medicine.

## Methods

### Sex as a biological variable.

This study was conducted in male rats to avoid the potential influence of the hormonal cycle on the regenerative process in female rats.

### Animals.

Male *Lewis* rats (SLC, Hamamatsu, Japan), weighing 300–400 g, were used for all experiments. The rats were maintained on a standard laboratory diet and water ad libitum. Temperature and humidity were controlled under a constant 12-hour light/dark cycle in the animal facility at Kyoto University.

### Preparation of intestinal decellularization.

Under general anesthesia with isoflurane, a midline abdominal incision was made to expose the abdominal cavity. The SMV was carefully isolated, and its small intestinal and colorectal tributaries were ligated and transected. Following systemic administration of heparin (0.5 U/g body weight), a 24-gauge cannula was inserted into the abdominal aorta. After transecting the inferior vena cava, 50 mL of phosphate-buffered saline (PBS) was perfused through the aortic cannula. An additional 22-gauge cannula was then inserted into the SMV. The small intestine was divided 3 cm distal to the ligament of Treitz and at the terminal ileum, with associated marginal vessels ligated accordingly. The SMA was subsequently dissected, and the entire small intestine was excised. The luminal contents were flushed thoroughly with PBS. The excised small intestine was placed in a PBS-filled culture dish and stored at –20°C overnight.

### DNA quantification.

Residual DNA content in native and decellularized intestinal tissues was measured using a DNA extraction kit (NucleoSpin Tissue, Macherey-Nagel, 740952.50), and quantified with a spectrophotometer (NanoDrop 2000, Thermo Fisher Scientific). DNA concentration was normalized to tissue dry weight (ng/mg).

### Histological analysis.

H&E staining and immunofluorescence staining were performed as previously described (26). Tissue specimens were fixed with 4% paraformaldehyde, embedded in paraffin, and cut into sections (thickness: 4 μm). For immunofluorescence, specimens were deparaffinized, autoclaved, and blocked. Sections were incubated with primary antibodies overnight at 4°C and secondary antibodies for 1 hour at room temperature. Native intestine samples were obtained from the ileum of naive rats. The primary and secondary antibodies are listed in Supplemental Table 1. αSMA monoclonal antibody (Progen Biotechnik GmbH, 61001), βIII tubulin (Abcam, ab18207), CDX2 antibody (Abcam, ab76541), Chromogranin A polyclonal antibody (Proteintech, 10529-1-AP), Collagen 1 polyclonal antibody (Bioss, bs-10423R), E-cadherin polyclonal antibody (Progen Biotechnik GmbH, 20874-1-AP), GLUT2 polyclonal antibody (Proteintech, 20436-1-AP), Human/Mouse/Rat CD31/PECAM-1 antibody (R & D Systems, AF3628), Ki67 antibody (Abcam, ab16667), Laminin polyclonal antibody (Bioss, bs-0821R), LGR5 polyclonal antibody (Invitrogen, PA5-87974), Lyve1 polyclonal antibody (OriGene, DP3513P), MUC2 polyclonal antibody (Proteintech, 27675-1-AP), SGLT1 polyclonal antibody (Proteintech, 30861-1-AP), Sox9 antibody (Merck Millipore, AB5535), Sucrase-isomaltase antibody (Santa Cruz Biotechnology, sc-393470), VEGF (Santa Cruz Biotechnology, sc-507), Vimentin (Dako, M0725), Villin polyclonal antibody (Proteintech, 16488-1-AP), ProLong Gold Antifade Mountant with DNA Stain DAPI (Invitrogen, P36931), Alexa 488-conjugated anti-rabbit IgG (Invitrogen, A-11008), Alexa 555-conjugated anti-mouse IgG (Invitrogen, A-31570), and Alexa 594-conjugated anti-mouse IgG (Invitrogen, A-11032). All sections were imaged using a BZ-9000 microscope (Keyence).

### Blood flow analysis.

Blood flow was visualized using an ICG fluorescence system (PDE-neo-System, Hamamatsu Photonics K.K.). The camera was fixed at 15 cm above the regenerated intestine in a dark room. ICG (0.2 mL of a 5 mg/mL) was injected into the penile vein and the image was recorded (59, 60).

### In vivo absorption test.

The absorption ability of the regenerated intestines was evaluated 4 weeks after RITx. After clamping the anastomotic sites, 850 μM FHA and 25% glucose were injected into the regenerated intestines. Blood samples were collected before injection and 5 and 30 minutes after injection. The FHA concentration was quantified using a SpectraMax 340PC (Molecular Devices) at 481 nm (ex)/520 nm (em). This medium-chain fatty acid was absorbed via passive diffusion from the gastrointestinal tract to the capillaries without passing through the lymph vessels (13, 35). The glucose concentration was quantified using a glucose assay kit (LabAssay Glucose, FUJIFILM Wako Pure Chemical Corporation, 291-94001). After 5 minutes incubation at 37°C, the sample was read using SpectraMax 340PC at 505 nm (ex)/600 nm (em).

### Ex vivo absorption test.

An ex vivo analysis of regenerated intestines was performed 4 weeks post-RITx. After systemic heparinization (0.5 U/g body weight), the abdominal aorta was cannulated with an 18-gauge cannula, and 50 mL of cold PBS was perfused following the transaction of the inferior vena cava. Next, 20 gauge and 24 gauge cannulas were cannulated into the SMA and SMV. The regenerated intestine and its mesentery, including the SMA and SMV, were isolated and removed. The bowel lumen was flushed with PBS, and the regenerated intestinal graft was stored in a cell culture dish with 4°C PBS. The SMA of the regenerated intestinal graft was connected to a pump and perfused with oxygenated crystalloid solution (SOLULACT Infusion, TERUMO, TP-AB05NR) at a flow rate of 1.15 mL/min in a 37°C water bath. After incubation, 850 μM of FHA and 25% glucose were injected into the regenerated intestine. Venous effluent (0.5 mL) was collected 5 minutes after injection, and the time of collection was recorded (13, 61). The samples were analyzed as described above.

### Peristalsis assessment.

The native intestine, decellularized intestine, and regenerated intestines 4 weeks after DITx and after RITx were analyzed. After excising each intestinal graft, both ends of the intestine were secured to connectors in Hank’s balanced salt solution (HBSS) at 37°C. Subsequently, 1 mL of HBSS was gently injected into the lumen of the graft to distend the bowel, and peristaltic movements were recorded for 60 seconds without pharmacological stimulants (26, 62). Acceptable peristaltic activity was observed immediately after tissue excision and captured for 60 seconds. The horizontal movement of a point marked at the center of the intestinal tract was analyzed using ImageJ (NIH) to create a time-displacement plot representing rhythmic displacement over time rather than a full spatiotemporal map (63–65).

### Evaluation of the nutritional status post-RITx in the rat SBS model.

We investigated the nutritional status in the following 3 groups: the RITx group, in which the whole native intestine was resected and 3 cm of regenerated intestine 4 weeks after DITx was anastomosed between the duodenum and terminal ileum; the SBS group, the whole native intestine was resected and the duodenum was anastomosed to the terminal ileum; and the control group, 3 cm of native intestine was left and the other intestine was resected. A 5 mm terminal ileum with an ileocecal valve was preserved, as previously reported (15). All surgical procedures in each model were performed under general anesthesia with isoflurane. Intestinal anastomoses were performed by running sutures with 6-0 VICRYL. The rats were allowed ad libitum access to a standard laboratory diet and water after the surgery. Body weight was measured in every morning, and blood tests were performed on POD 28. Changes in body weight loss rate (body weight/body weight on POD 0), and blood test parameters were compared among the groups.

### GLP-2 measurement.

Serum GLP-2 concentrations were measured using an enzyme immunoassay kit (Rat GLP-2 ELISA Kit, Yanaihara Institute Inc., YK140) according to the manufacturer’s protocol.

### DITx in SBS model.

Based on our DITx model, we conducted DITx in the SBS model (Supplemental Figure 6A). Approximately 1 cm of the terminal ileum was preserved, and the remaining distal small intestine was resected to induce SBS.

### Statistics.

Data are expressed as the mean ± SEM. All statistical analyses were performed using the JMP version 16.1 (SAS Institute). *P* < 0.05 was considered to indicate statistical significance. Two-tailed Student’s *t* test was used to compare 2 independent groups. Multiple groups were compared using 1-way ANOVA followed by Tukey’s honestly significant difference (HSD) test. The data of in vivo absorption tests were analyzed using a linear mixed-effects model with group, time, and their interaction as fixed effects, and individual rats as random effects. An autoregressive covariance structure [AR(1)] was applied to account for repeated measures. Postoperative body weight loss rates were also analyzed using a linear mixed-effects model with the same fixed and random effect structure. Tukey’s HSD test was used for post hoc pairwise comparisons.

### Study approval.

All animal experiments followed the ARRIVE guidelines and were approved by the Animal Experimentation Committee of Kyoto University (approval no. Med kyo 24520) and performed in accordance with the Animal Protection Guidelines of Kyoto University.

### Data availability.

All underlying data for the figures and statistical analyses presented in this manuscript have been provided as Supporting Data Values. This study did not generate or utilize any custom analytic code or software algorithms, and therefore, code availability is not applicable. Any additional raw data are available from the corresponding author upon reasonable request, subject to institutional regulations and approval from the IRB of Kyoto University Hospital.

## Author contributions

KI, TI, and HK participated in this study. KI, TI, and HK participated in the writing of the manuscript. KI performed experiments. KI, TI, HK, FM, HH, KM, TK, YH, EYU, SO, and EH participated in data analysis. All authors contributed to the interpretation of the data. All authors revised the manuscript and approved its final version.

## Conflict of interest

The authors have declared that no conflict of interest exists.

## Funding support

- Japan Society for the Promotion of Science (JSPS) KAKENHI, Grant no, 24K11864 to TI, 24K19356 to HK, and 26K11502 to EU

## Supplementary Material

Supplemental data

Supplemental video 1

Supplemental video 2

Supporting data values

## Version 1 06/03/2026

In-Press Preview

## Version 2 07/22/2026

Electronic publication
