# Supplementary material for: Autogenic-regenerated intestinal transplantation improves outcomes in short bowel syndrome
Source: JCI Insight. 2026 Jun 2;11(14):e200275. doi: 10.1172/jci.insight.200275 (PMC13461195; doi:10.1172/jci.insight.200275)
Supplement: Supplementary file 1 [file jciinsight-11-200275-s064.pdf]

**SUPPLEMENTAL CONTENT:**

**Autogenic-regenerated intestinal transplantation improves outcomes in short bowel syndrome**

**AUTHORS:**

Kentaro Iwaki, M.D., Takamichi Ishii, M.D., Ph.D., Hidenobu Kojima, M.D., Ph.D., Fumiaki Munekage, M.D., Hiroshi Horie, M.D., Kenta Makino, M.D., Ph.D., Takuma Karasuyama, M.D., Yusuke Hanabata, M.D., Elena Yukie Uebayashi, M.D., Ph.D., Satoshi Ogiso, M.D., Ph.D., Etsuro Hatano, M.D., Ph.D.

# Supplemental Table 1. List of primary and secondary antibodies used

Antibodies used

| Name | Host | Dilution | Cat# | Provider |
|---|---|---|---|---|
| Primary antibodies | | | | |
| Alpha-smooth muscle actin monoclonal antibody | Mouse | 1:1 | 61001 | Progen Biotechnik GmbH, Heidelberg, Germany |
| βIII tubulin | Rabbit | 1:2000 | ab18207 | Abcam, Cambridge, UK |
| CDX2 antibody [EPR2764Y] | Rabbit | 1:250 | ab76541 | Abcam, Cambridge, UK |
| Chromogranin A polyclonal antibody | Rabbit | 1:200 | 10529-1-AP | Proteintech, Rosemont, IL, USA |
| Collagen 1 polyclonal antibody | Rabbit | 1:50 | bs-10423R | Bioss, Boston, MA, USA |
| E-cadherin polyclonal antibody | Rabbit | 1:200 | 20874-1-AP | Progen Biotechnik GmbH, Heidelberg, Germany |
| GLUT2 polyclonal antibody | Rabbit | 1:200 | 20436-1-AP | Proteintech, Rosemont, IL, USA |
| Human/Mouse/Rat CD31/PECAM-1 antibody | Goat | 1:100 | AF3628 | R & D Systems, Minneapolis, MN, USA |
| Ki67 antibody | Rabbit | 1:100 | ab16667 | Abcam, Cambridge, UK |
| Laminin polyclonal antibody | Rabbit | 1:200 | bs-0821R | Bioss, Boston, MA, USA |
| LGR5 polyclonal antibody | Rabbit | 1:100 | PA5-87974 | Invitrogen, Waltham, MA, USA |
| Lyve1 polyclonal antibody | Rabbit | 1:50 | DP3513P | OriGene, Rockville, MD, USA |
| MUC2 polyclonal antibody | Rabbit | 1:100 | 27675-1-AP | Proteintech, Rosemont, IL, USA |
| SGLT1 polyclonal antibody | Rabbit | 1:400 | 30861-1-AP | Proteintech, Rosemont, IL, USA |
| Sox9 antibody | Rabbit | 1:200 | AB5535 | Merck Millipore, Burlington, MA, USA |
| Sucrase-isomaltase antibody (C-8) | Mouse | 1:50 | sc-393470 | Santa Cruz Biotechnology, Dallas, TX, USA |
| VEGF | Rabbit | 1:100 | sc-507 | Santa Cruz Biotechnology, Dallas, TX, USA |
| Vimentin | Mouse | 1:100 | M0725 | Dako (Agilent Technologies), Santa Clara, CA, USA |
| Villin polyclonal antibody | Rabbit | 1:100 | 16488-1-AP | Proteintech, Rosemont, IL, USA |
| ProLong™ Gold Antifade Mountant with DNA Stain DAPI | | 1:1 | P36931 | Invitrogen, Waltham, MA, USA |
| Secondary antibodies | | | | |
| Alexa 488-conjugated anti-goat IgG | Donkey | 1:500 | A-11055 | Invitrogen, Waltham, MA, USA |
| Alexa 488-conjugated anti-rabbit IgG | Goat | 1:500 | A-11008 | Invitrogen, Waltham, MA, USA |
| Alexa 555-conjugated anti-mouse IgG | Donkey | 1:500 | A-31570 | Invitrogen, Waltham, MA, USA |
| Alexa 594-conjugated anti-mouse IgG | Goat | 1:500 | A-11032 | Invitrogen, Waltham, MA, USA |

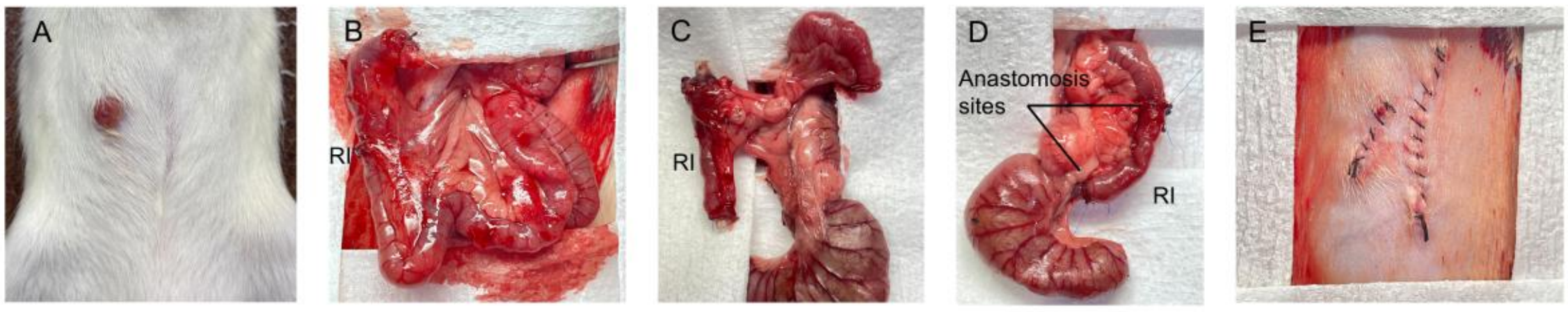


**Supplemental Figure 1. Intraoperative images of RITx.**

(**A**) Rat abdomen at 4 weeks post-DITx. (**B**) Re-laparotomy, jejunostomy closure, and dissection adhesions. (**C**) Isolation of the regenerated intestine while preserving its mesentery. The marginal arteries and veins at both ends of the regenerated intestine were ligated and dissected. (**D**) Re-anastomosis of the regenerated intestine to the jejunum and ileum. Both ends of the regenerated intestine were marked with nonabsorbable sutures. (**E**) Postoperative abdomen.

DITx, decellularized intestine transplantation; RI, regenerated intestine; RITx, regenerated intestine transplantation.

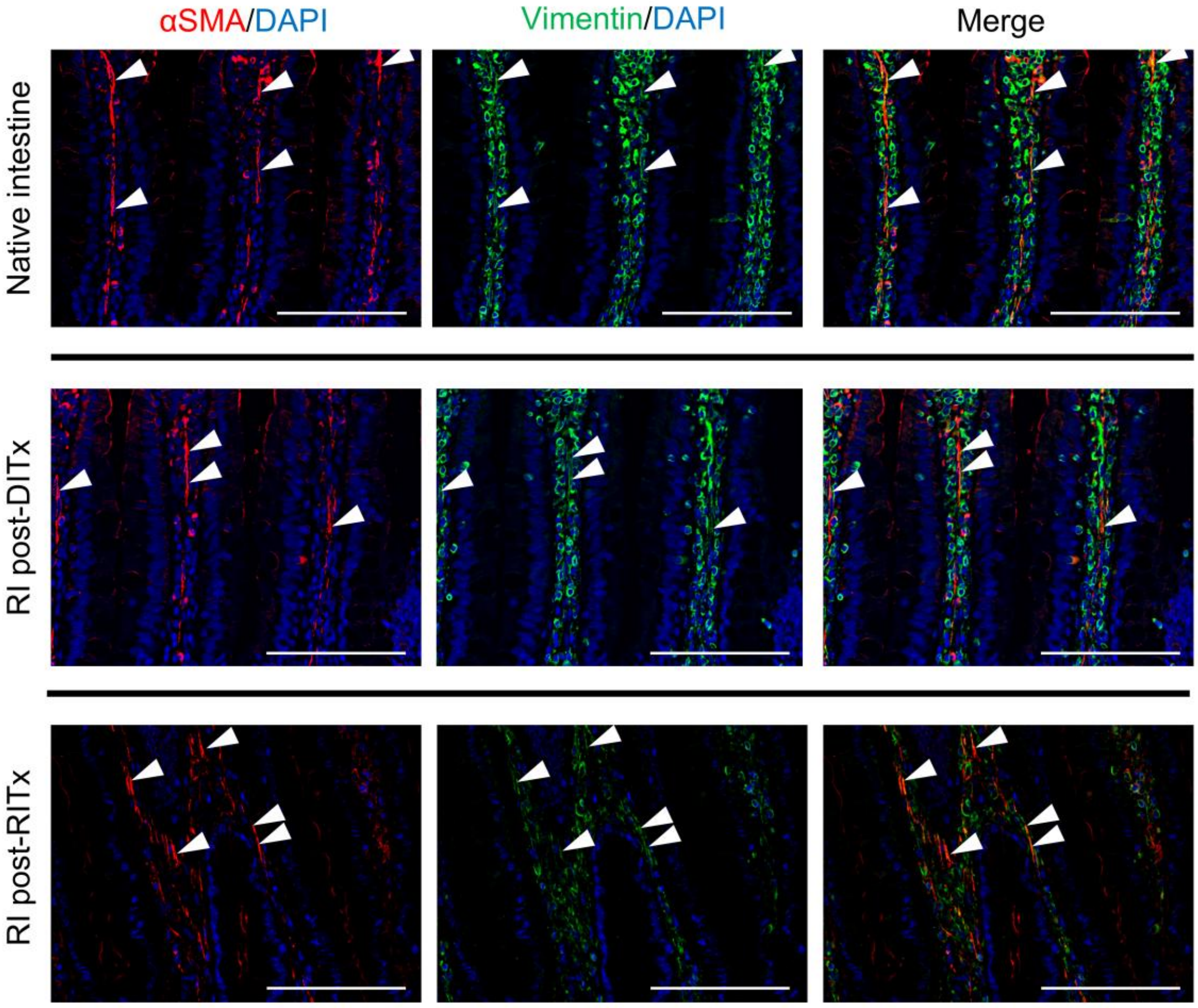


**Supplemental Figure 2. Immunofluorescence images of vimentin.**

Immunofluorescence images showing the expression of αSMA and vimentin in native and regenerated intestines post-DITx and RITx. Scale bar = 100 μm.

αSMA, α-smooth muscle actin; DAPI, 4′,6-diamidino-2-phenylindole; DITx, decellularized intestine transplantation; RI, regenerated intestine; RITx, regenerated intestine transplantation.

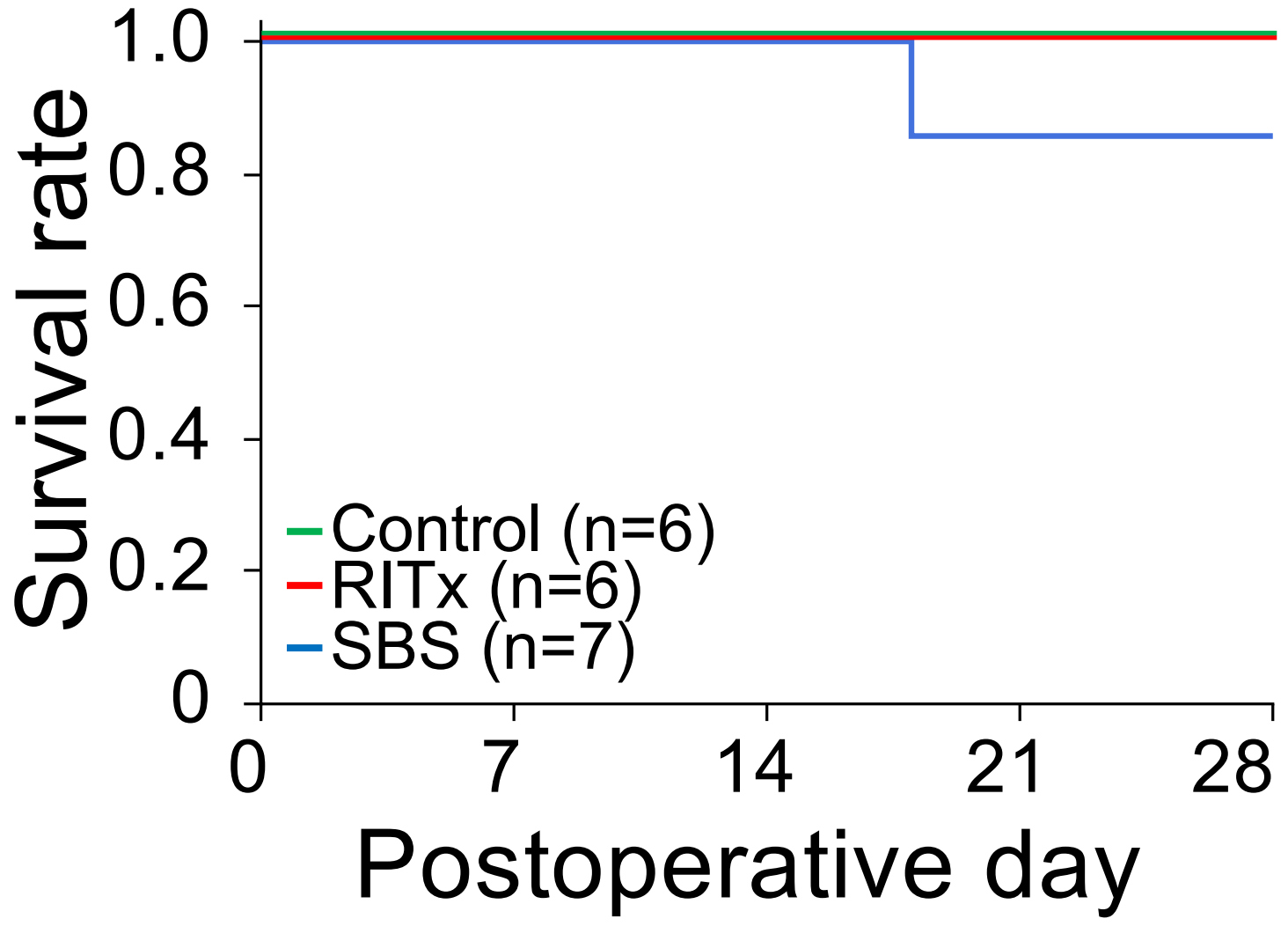


**Supplemental Figure 3. Survival curves of three models.**

Rats in control (green) and RITx (red) groups survived during the observation period.

One rat in the SBS (blue) group died on postoperative day 18.

RITx, regenerated intestinal transplantation; SBS, short bowel syndrome.

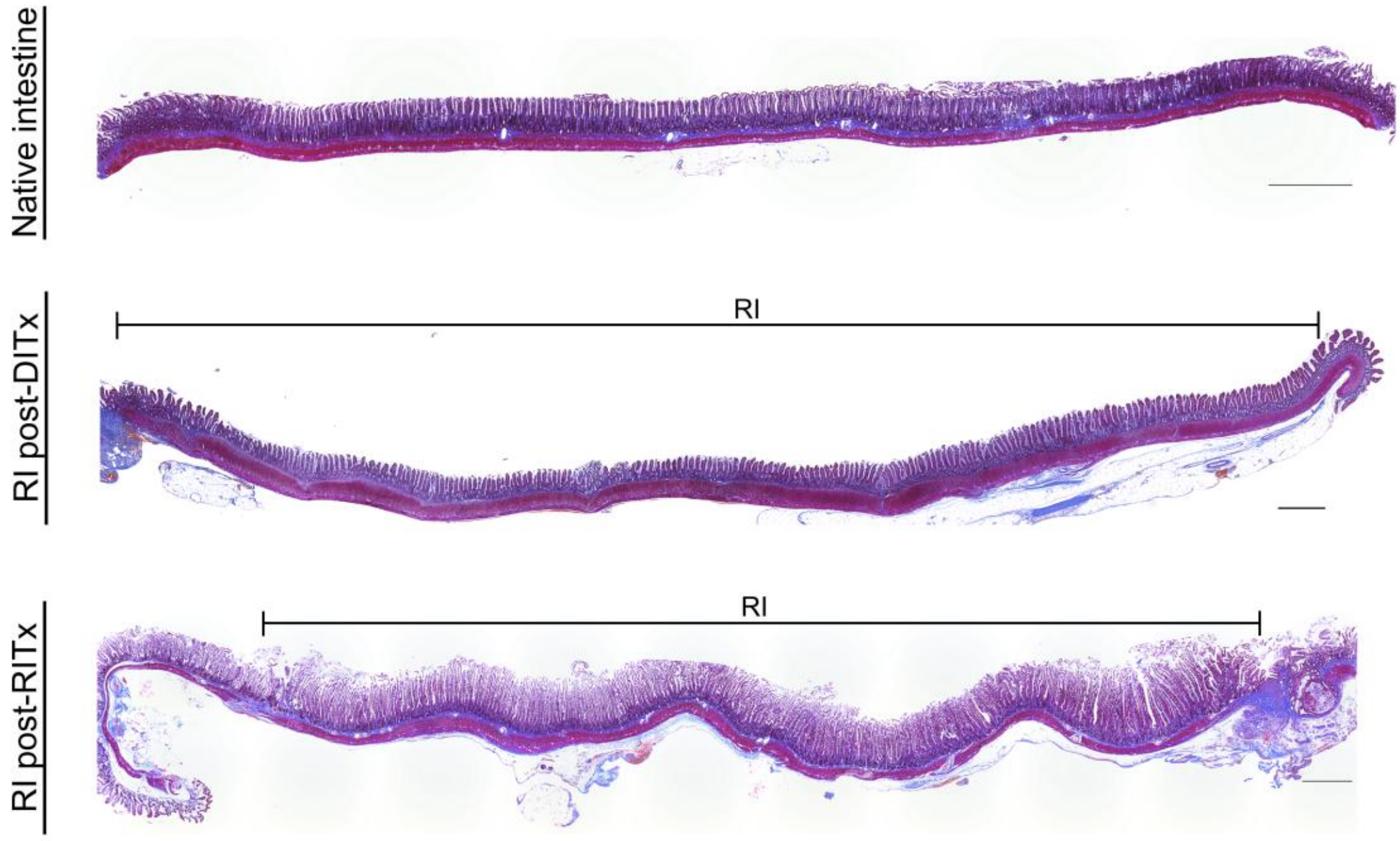


**Supplemental Figure 4. Masson's trichrome staining of regenerated intestines.**

Masson's trichrome staining of native and regenerated intestines. Scale bar = 1000 μm. DITx, decellularized intestine transplantation; RI, regenerated intestine; RITx, regenerated intestine transplantation.

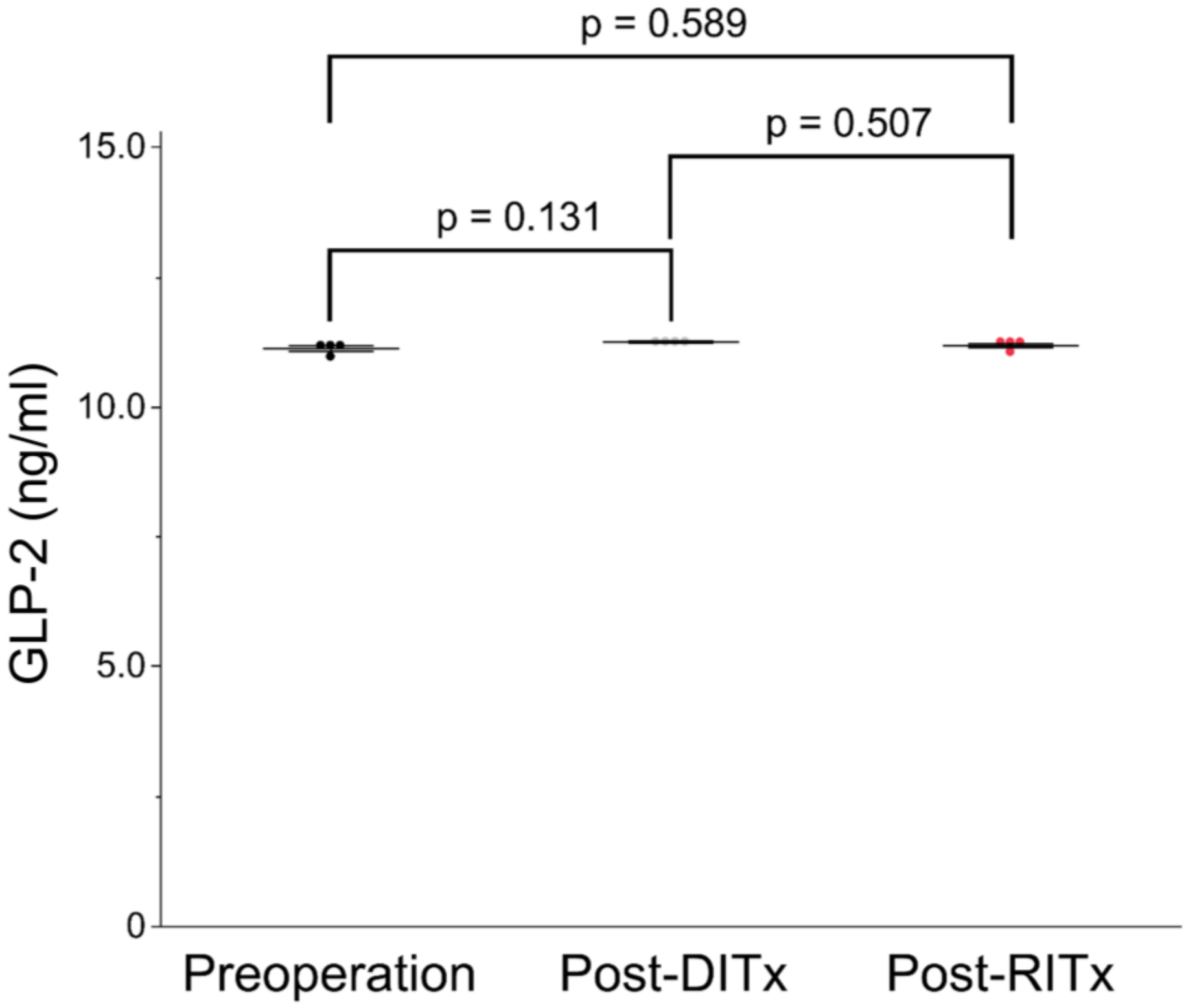


**Supplemental Figure 5. GLP-2 levels during the regeneration process.**

GLP-2 levels in rat serum of preoperation, 4weeks post-DITx, and 4 weeks post-RITx groups. n = 4 for each group. One-way ANOVA followed by Tukey's HSD test.

DITx, decellularized intestine transplantation; GLP-2, glucagon-like peptide-2; RITx, regenerated intestine transplantation.

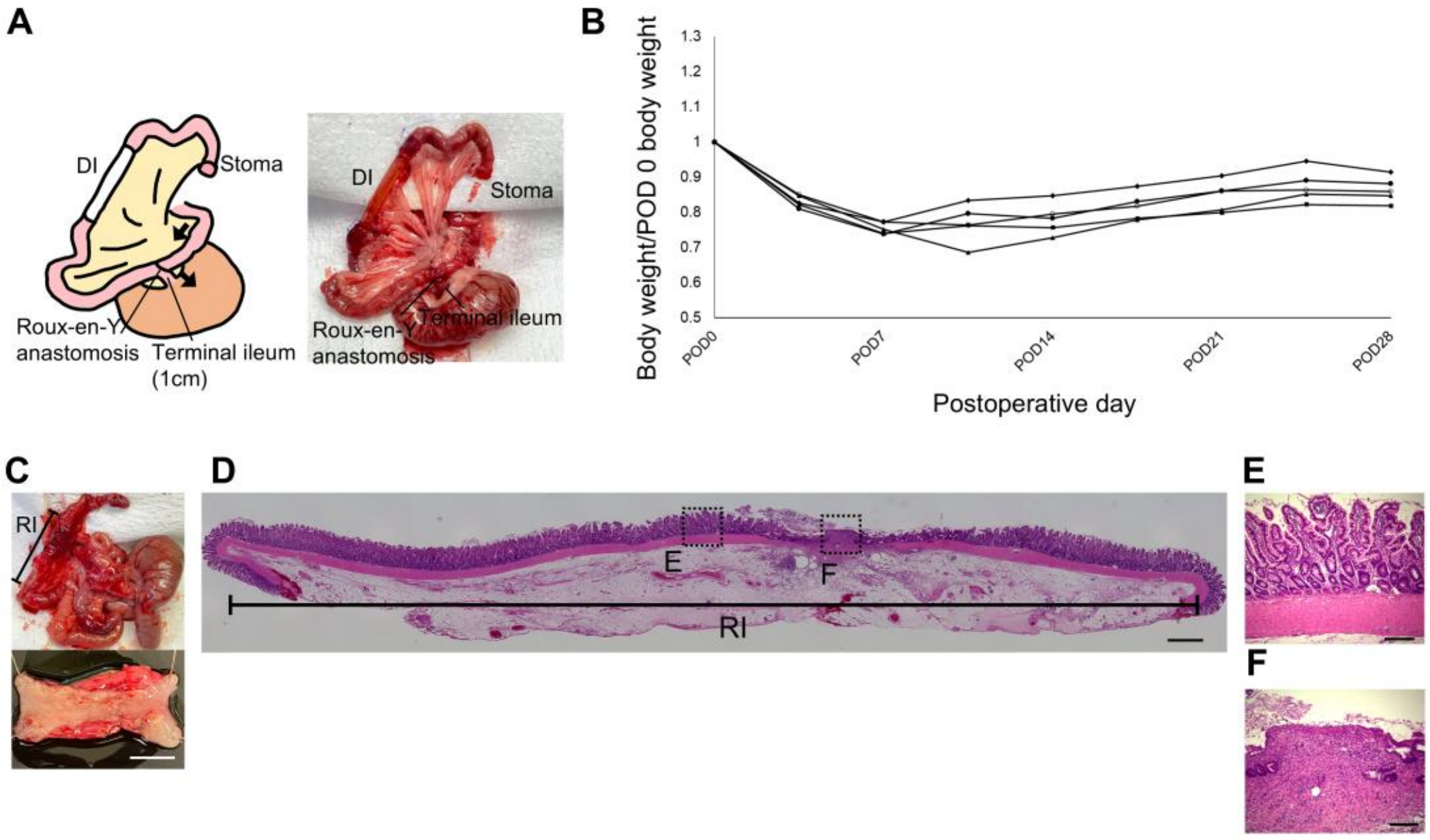


**Supplemental Figure 6. Intestinal regeneration in DITx with SBS model.**

(A) Schema of DITx in SBS rats. (B) Changes in the body weight loss rate (body weight/body weight on POD 0) after DITx in SBS. (C) Macroscopic images of the regenerated intestine 4 weeks post-DITx in SBS. Scale bar = 1 cm. (D) HE staining of the regenerated intestine 4 weeks post-DITx in SBS. Scale bars = 1000 µm. (E) High-magnification HE staining of regenerated area. Scale bar = 200 µm. (F) High-magnification HE staining of non-regenerated area. Scale bar = 200 µm.

DI, decellularized intestine; DITx, decellularized intestinal transplantation; HE, hematoxylin and eosin; POD, postoperative day; RI, regenerated intestine; SBS, short bowel syndrome.

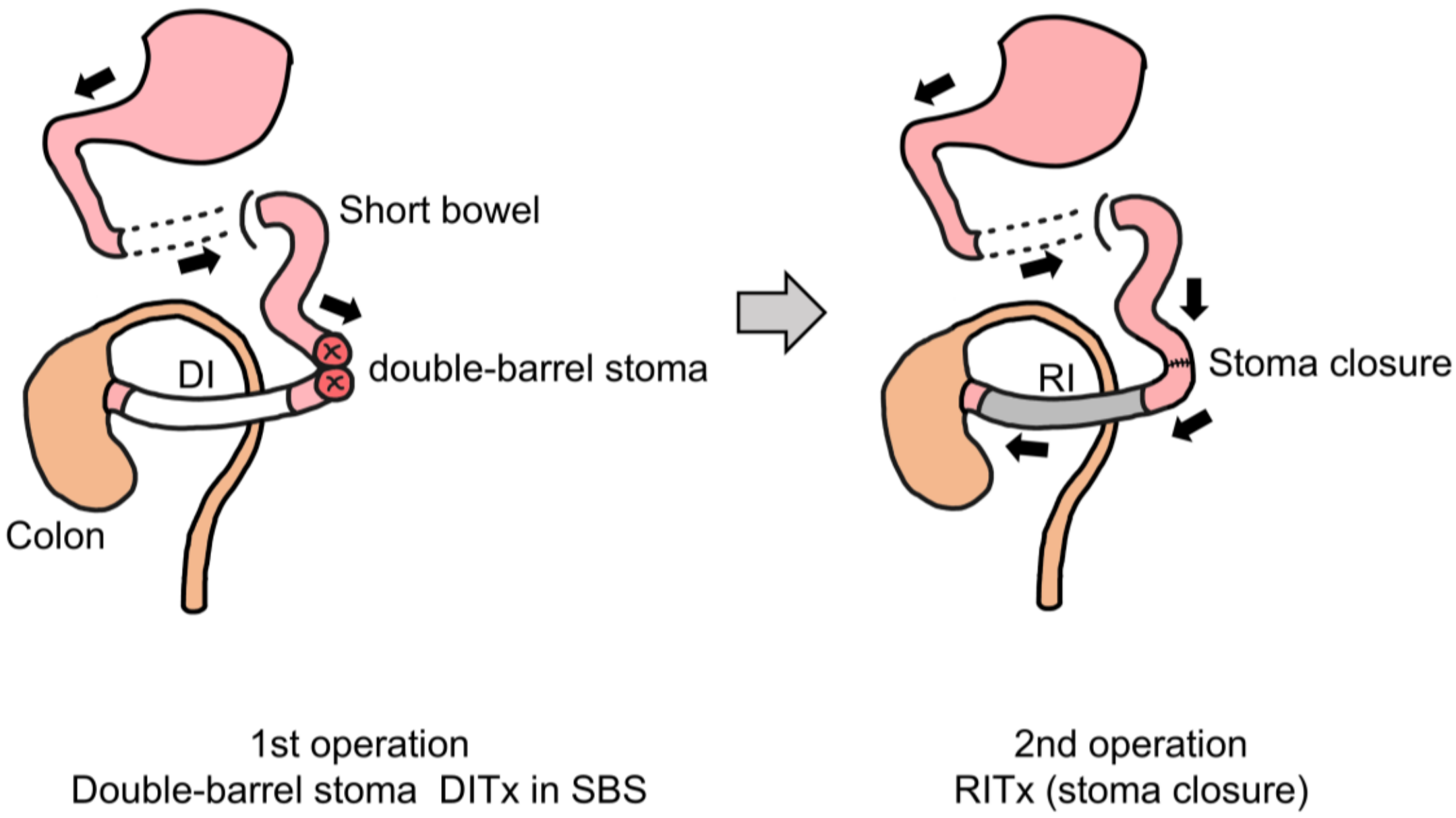


**Supplemental Figure 7. Schema of DITx model with a double-barrel stoma.**

The decellularized intestine is anastomosed to the distal limb of the double-barrel stoma. After regeneration, the regenerated intestine can be reconstructed in the alimentary tract by stoma closure.

DI, decellularized intestine; DITx, decellularized intestine transplantation; RI, regenerated intestine; RITx, regenerated intestine transplantation, SBS; short bowel syndrome.

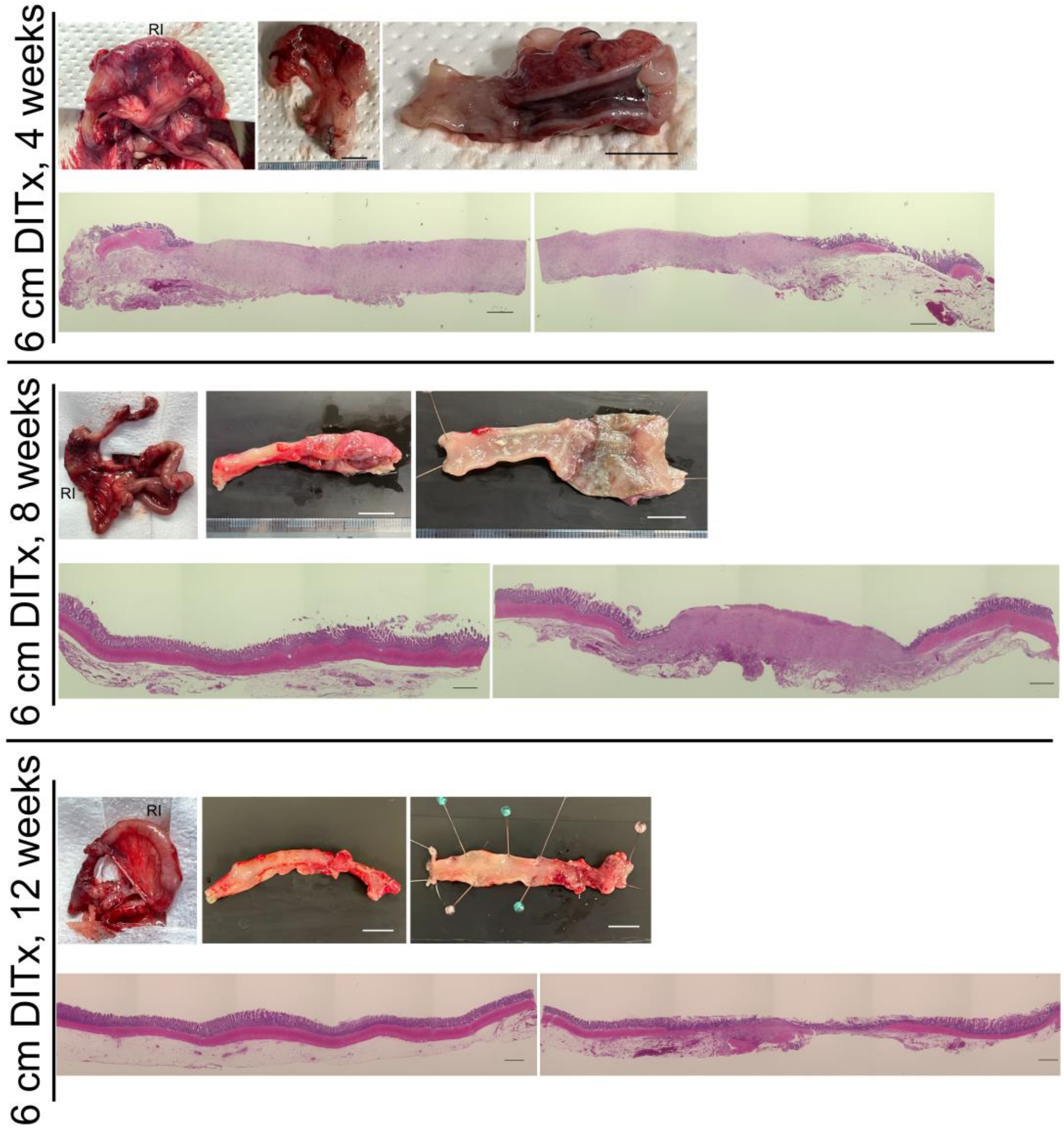


**Supplemental Figure 8. The intestinal regenerative process after 6cm-DITx.**

Macroscopic and microscopic images of the regenerated intestine at 4, 8, and 12 weeks post-DITx using a 6 cm decellularized intestinal scaffold. The two HE-stained images from each group were consecutive sections. Macroscopic images (upper panels), scale bar = 1 cm. HE staining images of the regenerated intestine (lower panels), scale bar = 1000 μm.

DITx, decellularized intestine transplantation; HE, hematoxylin and eosin; RI, regenerated intestine.

**Supplemental Video 1. Evaluations of blood flow in the regenerated intestine after DITx using an ICG fluorescence system.**

Evaluations of blood flow in the regenerated intestine 4 weeks post-DITx using an ICG fluorescence system. The first part of the video demonstrates blood flow in the regenerated intestine post-DITx following the injection of ICG after dissection of the adhesions. The second part shows the blood flow following the second injection of ICG after the isolation of the regenerated intestine post-DITx.

DITx, decellularized intestine transplantation; ICG, indocyanine green; RI, regenerated intestine.

**Supplemental Video 2. Evaluations of peristalsis.**

Peristaltic movements of the native intestine, decellularized intestine, and regenerated intestine post-DITx, and regenerated intestine post-RITx were recorded over 60 s.

DI, decellularized intestine; DITx, decellularized intestine transplantation; RI, regenerated intestine; RITx, regenerated intestine transplantation.
